# Type VI adenylyl cyclase negatively regulates GluN2B-mediated LTD and spatial reversal learning

**DOI:** 10.1038/srep22529

**Published:** 2016-03-02

**Authors:** Ching-Pang Chang, Cheng-Ta Lee, Wen-Hsien Hou, Meng-Syuan Lin, Hsing-Lin Lai, Chen-Li Chien, Chen Chang, Pei-Lin Cheng, Cheng-Chang Lien, Yijuang Chern

**Affiliations:** 1 Taiwan International Graduate Program in Molecular Medicine, National Yang-Ming University and Academia Sinica, Taipei, Taiwan; 2 Institute of Biomedical Sciences, Academia Sinica, Taipei, Taiwan; 3 Institute of Molecular Biology, Academia Sinica, Taipei, Taiwan; 4 Institute of Biochemistry and Molecular Biology, National Yang-Ming University, Taipei, Taiwan; 5 Institute of Neuroscience, National Yang-Ming University, Taipei, Taiwan

## Abstract

The calcium-sensitive type VI adenylyl cyclase (AC6) is a membrane-bound adenylyl cyclase (AC) that converts ATP to cAMP under stimulation. It is a calcium-inhibited AC and integrates negative inputs from Ca^2+^ and multiple other signals to regulate the intracellular cAMP level. In the present study, we demonstrate that AC6 functions upstream of CREB and negatively controls neuronal plasticity in the hippocampus. Genetic removal of AC6 leads to cyclase-independent and N-terminus of AC6 (AC6N)-dependent elevation of CREB expression, and enhances the expression of GluN2B-containing NMDA receptors in hippocampal neurons. Consequently, GluN2B-dependent calcium signaling and excitatory postsynaptic current, long-term depression, and spatial reversal learning are enhanced in the hippocampus of AC6^−/−^ mice without altering the gross anatomy of the brain. Together, our results suggest that AC6 negatively regulates neuronal plasticity by modulating the levels of CREB and GluN2B in the hippocampus.

Adenylyl cyclases (ACs) are a family of enzymes that convert ATP into adenosine 3′,5′ cyclic mononucleotide (cAMP), an important second messenger that controls cellular functions. The activity of ACs is regulated by G protein-coupled receptors (GPCRs) and various signaling molecules, including Ca^2+^, calmodulin, protein kinases, and nitric acid (NO)^1^. In total, 9 different membrane-bound ACs have been identified in mammals. Abnormal AC activity in neurons disturbs proper neuronal activity and leads to neuronal dysfunction. For example, deficiencies in AC1 and AC8 impair long-term potentiation (LTP) and memory, suggesting that AC1 and AC8 have important roles in the regulation of synaptic plasticity^2^. AC6 is a calcium-inhibited AC that is widely expressed in many brain areas at moderate levels^3^. We previously demonstrated that AC6 negatively modulates neurite outgrowth in hippocampal neurons and controls sympathetic tone in the brainstem^4^,^5^. The function of AC6 in the brain remains largely uncharacterized.

The N-methyl-D-aspartate receptor (NMDAR) is an ionotropic glutamate receptor that mediates many neuronal functions, including learning and memory. The activation of NMDARs triggers multiple signaling pathways that are critical for synaptic plasticity (i.e., LTP; long-term depression, LTD) and learning and memory^6^. There are three major types of NMDAR subunits (GluN1, GluN2A, and GluN2B) and these form NMDAR heteromers (e.g., GluN2A/GluN1, GluN2B/GluN1, or GluN2A/GluN2B/GluN1) with distinct functions in the hippocampus^7^. Alterations in the subunit composition of NMDAR severely impair synaptic plasticity^8^. We are particularly interested in the regulation of GluN2B by AC6 in the hippocampus because a cAMP response element-binding protein (CREB) binding site is located in the promoter region of GluN2B gene^46^ and GluN2B plays a critical role in controlling hippocampal functions during development^10^. Furthermore, the synaptic GluN2B level is regulated by synaptic activity in a CREB-dependent manner^11^. Finally, the CREB-dependent transcription of GluN2B contributes to the cocaine-induced locomotor sensitization^12^ and the action of ethanol^13^, further suggesting that CREB is a major regulator of GluN2B. In the present study, we present evidence to demonstrate that AC6 negatively modulates the CREB/GluN2B-mediated synaptic plasticity and LTD in the hippocampus and spatial reversal learning.

## Results

### AC6 exists in the hippocampus and functions upstream of CREB

**Figure 1:**
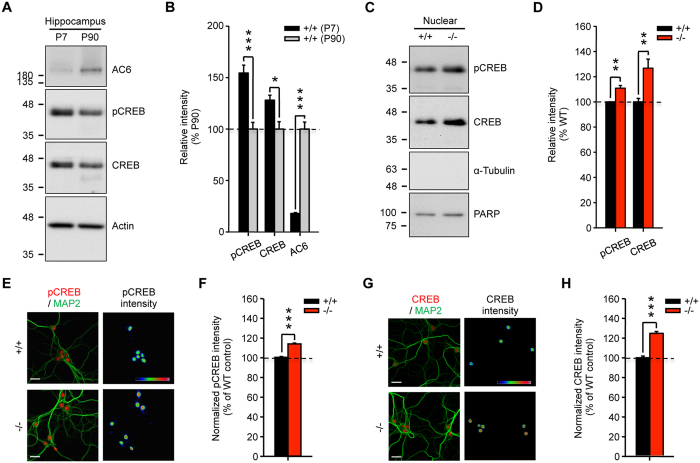
AC6 negatively regulates CREB level in the hippocampus. (**A**,**B**) Total lysate (Total, 20 μg) harvested from the hippocampi of postnatal day (P) P7 and P90 (n = 3–6) mice were subjected to WB analysis. (**A**) The expression level of AC6, pCREB, CREB, and actin were detected using the indicated antibody. Actin was used as a loading control. The level of the indicated protein was quantified, normalized with that of P90, and shown in (**B**). (**C**,**D**) Hippocampal nuclear fraction (5 μg) of AC6^+/+^ (n = 6) and AC6^−/−^ mice (n = 6) at P90 (3 months of age) were subjected to WB analysis. (**C**) The expression level of pCREB and CREB were detected using the indicated antibodies. PARP was used as the internal loading control of nuclear fraction. The protein expression level was quantified and is shown in (**D**). (**E–H**) Primary hippocampal neurons of AC6^+/+^ and AC6^−/−^ mice were fixed at DIV14 to analyze the levels of pCREB (red, **E**) and CREB (red, **G**) by immunostaining. (**E,G**) MAP2 (green) is a neuronal marker. The color bars in the upper-right panel, from cold to warm colors, represent low to high fluorescence intensities of pCREB or CREB as indicated. Scale bars, 25 μm. The intensity of pCREB (**F;** AC6^+/+^, n = 436; AC6^−/−^, n = 487) or CREB (**H**; AC6^+/+^, n = 157; AC6^−/−^, n = 139) in each cell was divided by the mean value of the AC6^+/+^ cells and is represented as the means ± SEM. Data were analyzed using Student’s *t*-test. *p < 0.05; **p < 0.01; ***p < 0.001, versus the control group.

We previously reported that the amount of AC6 protein increased in the brain during postnatal development, suggesting that AC6 may be involved in the maturation of hippocampus^5^. Given that CREB is a downstream target of ACs and has been implicated in the maturation of hippocampus and neuronal plasticity, we assessed the amounts of total CREB and activated CREB (phosphorylated CREB, pCREB) in young and adult hippocampus. As shown in Fig. 1A,B, the amounts of CREB and pCREB were inversely correlated with that of AC6 in the hippocampus at postnatal day 7 (P7) and P90. Conversely, genetic removal of AC6 in the hippocampus enhanced the level of CREB and pCREB (Fig. 1C,D), suggesting that CREB may function as a downstream target of AC6 in the adult hippocampus.

### AC6 negatively regulates the level of CREB through its N terminus and requires no cyclase activity

**Figure 2:**
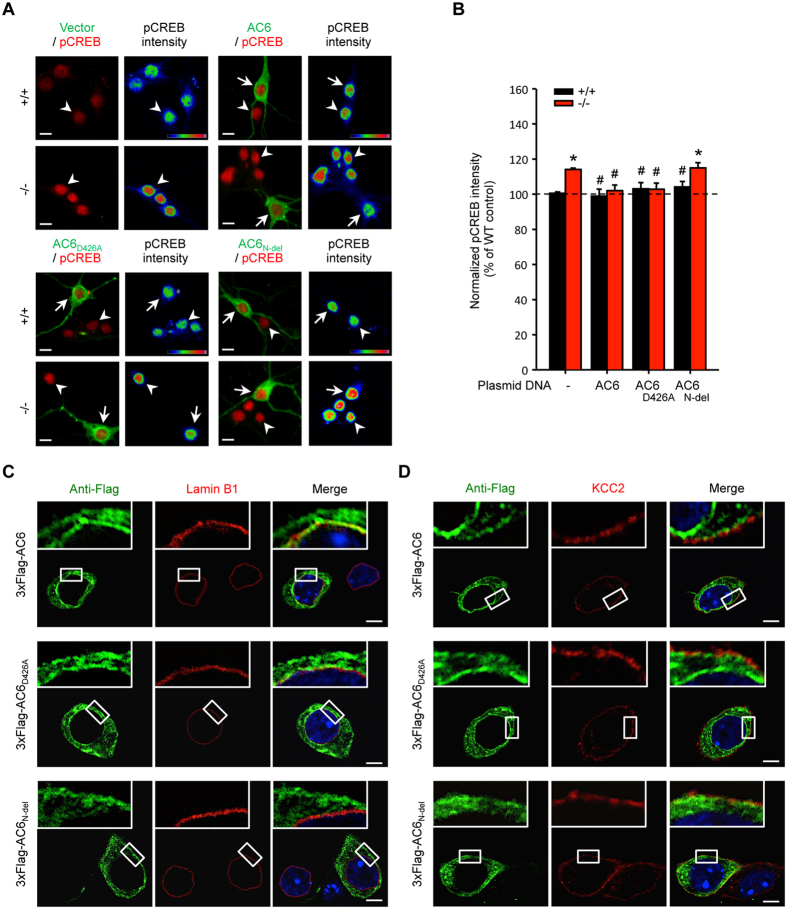
Exogenous expression of AC6 variants (AC6 and AC6_D426A_), which are located on the plasma and nuclear membranes, normalized the enhanced CREB activity in AC6^−/−^ hippocampal neurons. (**A**) Primary hippocampal neurons of AC6^+/+^ and AC6^−/−^ mice were transfected with 3xFlag-vector, 3xFlag-AC6, 3xFlag-AC6_D426A_, or 3xFlag-AC6_N-del_ at DIV10 and fixed to analyze the expression of anti-pCREB (red) and anti-flag (green) at DIV14 through immunocytochemical staining. The color bars in the panels labeled as “pCREB intensity” represent the level of pCREB intensity, from low to high fluorescence signals (blue → red). The arrows and arrowheads indicate the transfected and non-transfected primary hippocampal neurons. Scale bar, 10 μm. (**B**) The intensity of pCREB in each transfected cell was divided by the mean value of the non-transfected AC6^+/+^ cells and is represented as the means ± SEM (Relative pCREB intensity; non-transfected AC6^+/+^ cells, n = 436; non-transfected AC6^−/−^ cells, n = 487; 3xFlag-AC6- transfected AC6^+/+^ cells, n = 22; 3xFlag-AC6- transfected AC6^−/−^ cells, n = 24; 3xFlag-AC6_D426A_ transfected AC6^+/+^ cells, n = 20; 3xFlag-AC6_D426A_ transfected AC6^−/−^ cells, n = 17; 3xFlag-AC6_N-del_ transfected AC6^+/+^ cells, n = 20; 3xFlag-AC6_N-del_ transfected AC6^−/−^ cells, n = 18). The data represent the means ± SEM in each group. *p < 0.01, compared with AC6^+/+^ control neurons; ^#^*p* < 0.01, compared with AC6^−/−^ control neurons; one-way ANOVA. (**C,D**) The subcellular localization of exogenously expressed AC6, AC6_D426A_, or AC6_N-del_ (anti-Flag, green) in transfected AC6^+/+^ hippocampal neurons (DIV14) was evaluated after immunostaining with (**C**) the nuclear membrane marker (Lamin B1, red) and (**D**) the plasma membrane enriched protein (KCC2, red), respectively. Hoechst33258 (blue) was used as the nuclear marker. From top to bottom, the images represent 3xFlag-AC6, 3xFlag-AC6_D426A_, and 3xFlag-AC6_N-del_ transfected hippocampal neurons; scale bar, 5 μm.

Consistent with the above observation, the level of pCREB and CREB assessed by immunofluorescence in primary hippocampal neurons (DIV14) harvested from AC6 KO (AC6^−/−^) was higher than that of wild-type (AC6^+/+^) (Fig. 1E–H). To assess the underlying mechanism that mediates the suppression of CREB by AC6, we transfected various AC6 variants to primary hippocampal neurons. Exogenous expression of AC6 (3xFlag-AC6) or a cyclase-dead AC6 mutant (3xFlag-AC6_D426A_)^5^ normalized the amount of pCREB in AC6^−/−^ hippocampal neurons, but did not affect pCREB expression in AC6^+/+^ neurons (Fig. 2A,B). In contrast, exogenous expression of an N-terminal truncated AC6 mutant (3xFlag-AC6_N-del_) had no effect on the elevated pCREB level in AC6^−/−^ neurons (Fig. 2A,B). The N-terminus of AC6 (amino acids 2–86 of AC6, designated AC6N) is of great importance because it contains a regulatory domain that interacts with Snapin and modulates the catalytic activity of AC6^5^,^14^.

Given that transmembrane ACs are generally thought to exist in the plasma membrane^9^, we were surprised to find that exogenous expressed 3xFlag-AC6 and 3xFlag-AC6_D426A_ could be detected not only on the plasma membrane (identified by the expression of a plasma membrane-enriched protein, the K^+^/Cl^–^ co-transporter KCC2) but also at the nuclear envelope (identified by the expression of Lamin B1). Conversely, exogenous expressed 3xFlag-AC6_N-del_ was detected only on the plasma membrane (Fig. 2C,D), but not on the nuclear envelope. These observations suggest that genetic removal of AC6 led to an elevation of CREB and pCREB in hippocampal neurons in a manner that was cyclase-independent but required AC6N. In addition, AC6N may mediate the localization of AC6 on the nuclear membrane in hippocampal neurons.

### Genetic removal of AC6 selectively enhances hippocampus-dependent learning without affecting overall brain morphology

**Figure 3:**
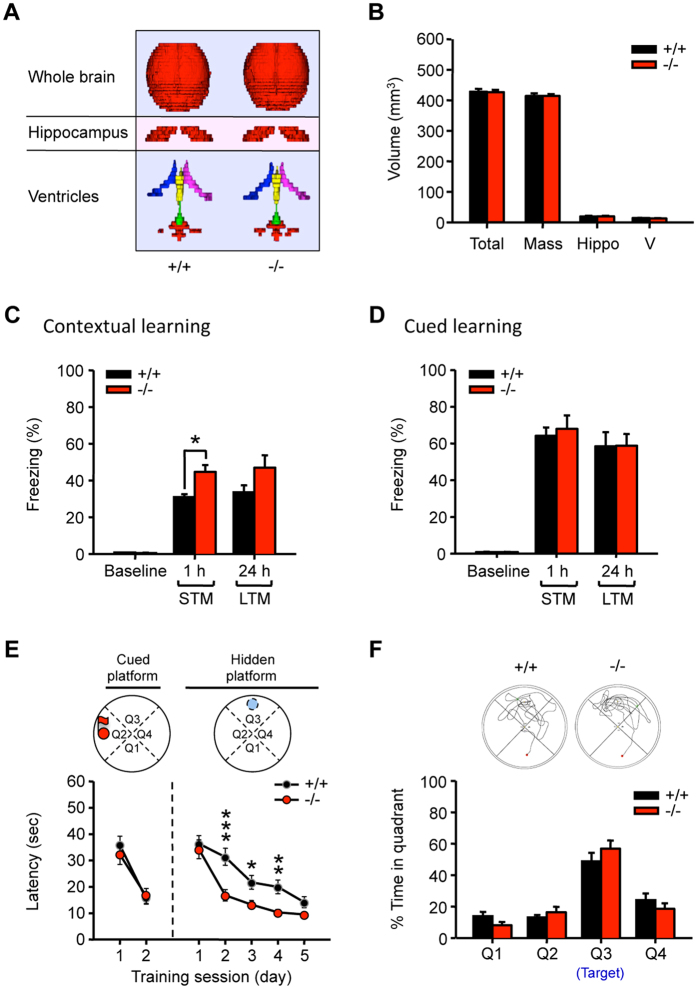
AC6^−/−^ mice had greater hippocampus-dependent learning ability than their littermate controls (AC6^+/+^). The mice used for the following experiments were 3–6 month-old. (**A**) Micro-MRI images of AC6^+/+^ and AC6^−/−^ mice. From top to bottom, the reconstituted images represented the whole brain, hippocampus, and ventricles (magenta: right lateral ventricle; blue: left lateral ventricle; yellow: 3rd ventricle; green: aqueduct; red: 4th ventricle). (**B**) The volume (mm^3^) of the brain (Total), hippocampus (Hippo), ventricles (V) and the brain mass of AC6^+/+^ (n = 5) and AC6^−/−^ (n = 3) mice were measured. (**C,D**) The freezing behavior of AC6^+/+^ (n = 6) and AC6^−/−^ (n = 8) mice was monitored. Both short-term (STM) and long-term memory (LTM) of contextual (**C**) and cued learning (**D**) were determined after measuring the percentage freezing time of the animals 1 and 24 hr, respectively, after receiving two white noise (90 dB)-foot shock (0.5 mA)-paired stimulations. (**B**–**D**) *p < 0.05; **p < 0.01; ***p < 0.001, versus the control littermates by Student’s test. (**E,F**) In the Morris water maze, the escape latencies to cued and hidden platforms of AC6^+/+^ (n = 9) and AC6^−/−^ (n = 7) mice were recorded for 2 and 5 days, respectively (**E**). Two-way ANOVA showed significant effects of genotype (F_(1,70)_ = 17.918, *p* < 0.001) and training day (F_(4,70)_ = 19.896, *p* < 0.001) with no interaction between these factors (F_(4,70)_ = 1.313, *p* = 0.274). *p < 0.05, **p < 0.01, ***, *p* < 0.001, by Fisher’s LSD post-hoc analysis. (**F**) For the probe trial, the percentage of time that AC6^+/+^ (n = 9) and AC6^−/−^ (n = 7) mice swam in each quadrant was measured. The target quadrant was Q3. Two-way ANOVA showed a significant effect of quadrant (F_(3,56)_ = 49.006, *p* < 0.001) but not genotype (F_(1,56)_ = 0.000001, *p* = 0.999), with no interaction between these factors (F_(3,56)_ = 1.565, *p* = 0.208). The data represent the means ± SEM in each group.

We previously reported that AC6 negatively regulates neurite extension in primary hippocampal neurons^5^. To assess whether the absence of AC6 affects hippocampal morphology, we examined overall brain structure of AC6^−/−^ mice using 3D micro MRI (Fig. 3A). The results showed that the brain volumes and the sizes of the hippocampus and ventricles of AC6^−/−^ and AC6^+/+^ mice were similar (Fig. 3B). The normal morphology of the hippocampus (including the CA1 and CA3 regions) of AC6^−/−^ mice was also verified using Nissl-staining (Supplementary Fig. 1A). We next investigated whether genetic ablation of AC6 could disrupt brain functions. We conducted a series of behavioral assays to assess motor function, pain sensation, anxiety, social behavior, and cognitive function. Both horizontal and vertical locomotor activities of AC6^−/−^ mice were similar to those of their littermate controls (Supplementary Fig. 1B). The latency to fall in the rotarod test showed no difference between AC6^−/−^ and AC6^+/+^ mice, suggesting that AC6^−/−^ mice have normal motor functions (Supplementary Fig. 1C). The open field test revealed that AC6^−/−^ mice had normal exploratory and anxiety-like behaviors (Supplementary Fig. 1D). In addition, AC6^−/−^ and AC6^+/+^ mice spent comparable amounts of time in the open and closed arms of the elevated plus maze (Supplementary Fig. 1E). Collectively, AC6^−/−^ mice showed normal anxiety-like behaviors.

We next employed the three-chamber sociability assay to evaluate the sociability of AC6^−/−^ mice. Because AC6^−/−^ and AC6^+/+^ mice spent similar amounts of time in a chamber containing a mouse that they had not met before (Stranger 1, Supplementary Fig. 1F, social affiliation) and both AC6^−/−^ and AC6^+/+^ mice spent more time in the chamber containing the “stranger” mouse (Stranger 2, Supplementary Fig. 1G, social preference), we concluded that AC6^−/−^ mice exhibited normal sociability.

Because previous studies demonstrated that multiple ACs (e.g., AC1, AC5, and AC8) are involved in the neuropathic pain pathway^15^, we therefore evaluated whether AC6 was involved in the pain response. Pain thresholds were determined by the von Frey assay, and the results showed that AC6^−/−^ and AC6^+/+^ mice had similar pain responses (Supplementary Fig. 1H).

Cognitive function was examined using the fear conditioning test and the Morris water maze. In the hippocampus-dependent contextual fear-conditioning test, freezing behavior was measured 1 and 24 hr after receiving two times of unconditioned stimuli (mild foot shock, 0.5 mA) paired with conditioned stimuli (white noise). Compared with AC6^+/+^ mice, the freezing level of AC6^−/−^ mice was significantly higher than that of AC6^+/+^ mice 1 hr after training (short-term memory) but not 24 hr after training (long-term memory) (Fig. 3C). In the amygdala-dependent cue learning test, the freezing level of AC6^−/−^ mice was similar to that of AC6^+/+^ mice (Fig. 3D). Collectively, AC6^−/−^ mice showed enhanced contextual, but not cued, memory. The hippocampus-dependent memory of AC6^−/−^ mice appeared superior to that of AC6^+/+^ mice.

Next, we assessed the spatial memory of AC6^−/−^ mice using the Morris water maze. During the pre-training session with a cued platform, there was no difference in the escape latency between AC6^−/−^ and AC6^+/+^ mice (Fig. 3E), indicating that AC6^−/−^ mice had normal visual function and hippocampus-independent memory^16^. During the 5-day training period with the hidden platform, the escape latency of AC6^−/−^ mice decreased significantly more than that of AC6^+/+^ mice from day-2 to day-4 (Fig. 3E), suggesting that AC6^−/−^ mice learned faster than AC6^+/+^ mice. Interestingly, AC6^−/−^ and AC6^+/+^ mice spent similar amounts of time in the target quadrant in the probe test (Fig. 3F). Once learned, the memory retention ability of AC6^−/−^ mice was similar to that of AC6^+/+^ mice. Consistent with the performance of these mice in the locomotor activity assay (Supplementary Fig. 1B), the swimming speeds of AC6^−/−^ and AC6^+/+^ mice were similar (Supplementary Fig. 1I). Collectively, AC6^−/−^ mice have higher hippocampus-dependent learning ability than AC6^+/+^ mice, but no change in the gross anatomy of the brain.

### AC6 negatively regulates the level of GluN2B-possessing NMDARs in the hippocampus

**Figure 4:**
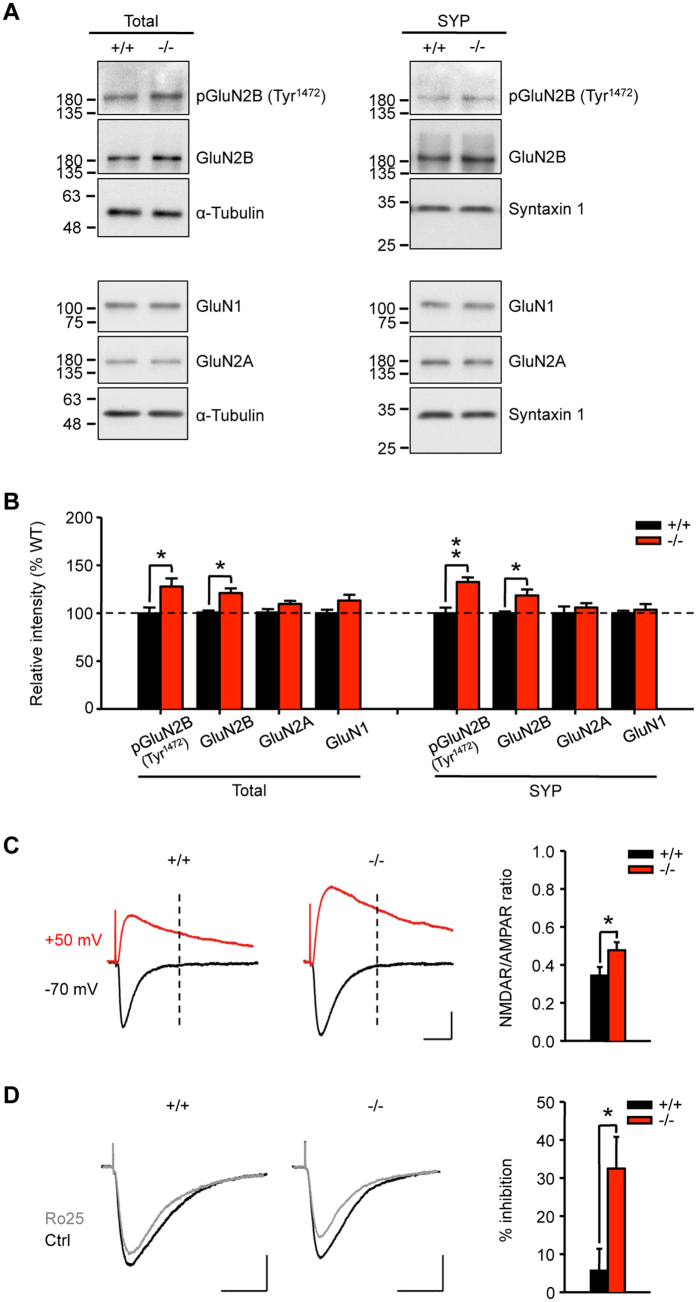
The GluN2B level and GluN2B-mediated EPSCs were elevated in the hippocampus of AC6^−/−^ mice. (**A**,**B**) Total hippocampal lysates (Total, 20 μg per lane) and synaptosome fractions (SYP, 5 μg per lane) of AC6^+/+^ (n = 3–5) and AC6^−/−^ mice (n = 3–5) at 3–6 months of age were subjected to WB analyses. (**A**) The expression levels of pGluN2B (Tyr^1472^), GluN2B, GluN2A, and GluN1 were detected using the indicated antibodies. α-Tubulin and syntaxin 1 were used as the internal loading controls for the total lysate and the synaptosome fraction, respectively. The protein expression level was quantified and is shown in (**B**). *p < 0.05; **p < 0.01, Student’s *t*-test. (**C**) The NMDAR/AMPAR ratio of the hippocampal slices from AC6^+/+^ mice (n = 17 slices from 6 mice) and AC6^−/−^ mice (n = 10 slices from 3 mice) at 3–6 months of age was obtained after measuring NMDAR- and AMPAR-mediated EPSCs at −70 mV and +50 mV, respectively. The vertical dashed lines indicate the measurement of NMDAR-mediated currents at +50 mV. The right-most panel shows the quantification of the NMDAR/AMPAR EPSC ratios. Scale bars: 50 pA, 20 ms. (**D**) The NMDAR-mediated EPSCs (−70 mV, in the presence of 10 μM CNQX) were recorded in the CA1 pyramidal neurons of AC6^+/+^ (n = 8 slices from 4 mice) and AC6^−/−^ (n = 6 slices from 3 mice) hippocampal slices from 3–6-month-old mice before and after treatment with Ro25-6981 (Ro25, 5 μM). The right-most panel shows the percentage of Ro25-mediated inhibition of the NMDAR-evoked EPSC. Scale bars: left, 50 pA, 50 ms; right, 100 pA, 50 ms. The data represent the means ± SEM of at least three independent experiments. (**C**,**D**) *p < 0.05, Wilcoxon rank-sum test.

Because GluN2B is a downstream target of CREB and plays a critical role in hippocampus-dependent learning and memory^11^, we next assessed the levels of GluN2B and other glutamate receptors (NMDA and AMPA receptor subunits) in the hippocampus. As shown in Fig. 4A, enhanced GluN2B expression was observed in the hippocampus of AC6^−/−^ mice. WB analysis revealed that the protein expression of GluN2B, but not other subunits of NMDAR (i.e., GluN2A and GluN1), was elevated in the total lysate and synaptosome fractions of the hippocampus (Fig. 4A,B). No alteration in the expression of two AMPA receptor subunits (GluA1 and GluA2) was observed in either the total lysate or the synaptosome fraction of the AC6^−/−^ hippocampus (Supplementary Fig. 2A,B). In addition, the transcript levels of GluN2B, but not GluA1, were elevated in the hippocampus of AC6^−/−^ mice (Supplementary Table 1).

Because phosphorylation of GluN2B and GluA1 is important for neuronal plasticity and learning and memory, we next determined the level of phosphorylation of GluN2B at Tyr^1472^ [designated pGluN2B (Tyr^1472^)]. In the hippocampus of AC6^−/−^ mice, the pGluN2B (Tyr^1472^) levels were elevated in both the total lysate and synaptosome fraction (Fig. 4A,B). This finding is important because the increased expression of pGluN2B (Tyr^1472^) is associated with trafficking and membrane expression of GluN2B-containing NMDARs^17^. No change in the levels of pGluA1 (Ser^845^) was observed between AC6^−/−^ and AC6^+/+^ mice (Supplementary Fig. 2A,B).

Consistent with our hypothesis that the hippocampal neurons of AC6^−/−^ mice had higher levels of GluN2B-containing NMDARs than AC6^+/+^ neurons, the ratio of NMDAR-mediated to AMPAR-mediated EPSCs in the CA1 pyramidal neurons of AC6^−/−^ mice was higher than that of AC6^+/+^ mice (AC6^+/+^, 0.34 ± 0.05 vs. AC6^−/−^, 0.47 ± 0.04; *p < 0.05, Fig. 4C). Moreover, the inhibitory effect of an GluN2B antagonist Ro25-6981 (5 μM)^18^ on NMDA receptor-mediated currents was greater in the CA1 pyramidal neurons of AC6^−/−^ mice than in those of AC6^+/+^ littermate controls (AC6^+/+^, 5.65 ± 5.76% vs. AC6^−/−^, 32.46 ± 8.33%; *p < 0.05, Fig. 4D). Ro25-6981 is a highly potent inhibitor of NMDA receptors containing the GluN2B subunit and has a >5,000-fold selectivity over those containing the GluN2A subunit^19^. Our results suggest that the absence of AC6 alters the NMDAR/AMPAR ratio by increasing the expression of GluN2B in the hippocampus.

**Figure 5:**
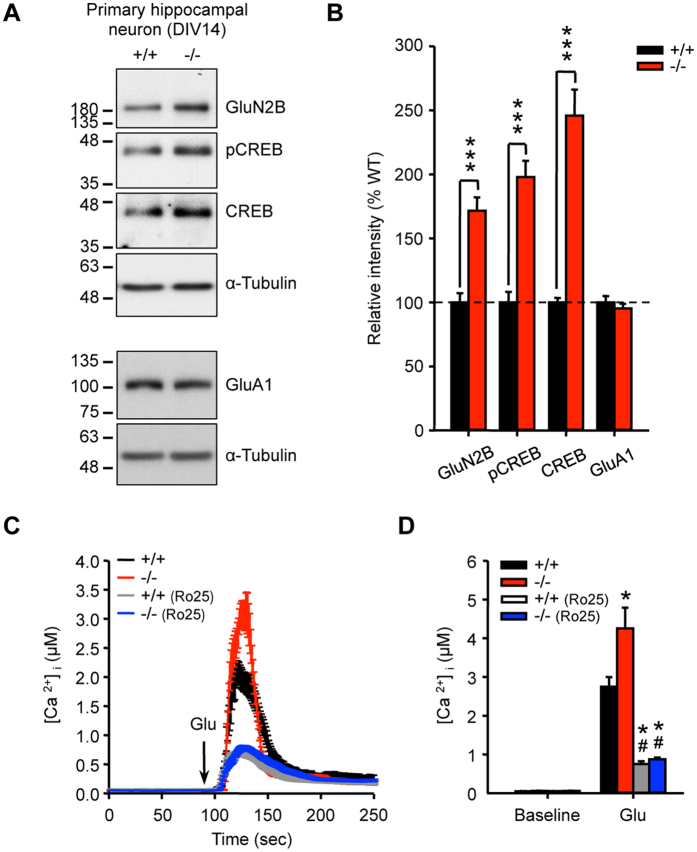
AC6^−/−^ hippocampal neurons contained more CREB and exhibited a higher GluN2B-mediated signal than AC6^+/+^ controls. (**A**,**B**) Total lysates of primary hippocampal neurons were harvested and subjected to WB analyses (8 μg per lane; AC6^+/+^, n = 3; AC6^−/−^, n = 4). (**A**) The expression levels of GluN2B, GluA1, pCREB, and CREB were detected using the indicated antibodies. α-Tubulin was used as the internal loading control. The quantification is shown in (**B**). ***p < 0.01, Student’s *t*-test. (**C**,**D**) Cytoplasmic calcium activity was evaluated in fura-2-loaded primary hippocampal neurons (DIV14). These neurons were pre-incubated with (AC6^+/+^, n = 39, grey; AC6^−/−^, n = 41, blue) or without (AC6^+/+^, n = 31, black; AC6^−/−^, n = 32, red) an GluN2B antagonist, Ro25-6981 (Ro25, 5 μM) at 37 °C for 30 min. (**C**) L-glutamate (Glu, 10 μM) and glycine (10 μM) were added to the medium as indicated to enhance the intracellular calcium concentration ([Ca^2+^]_i_). Basal and the maximum cytosolic Ca^2+^ responses evoked through L-glutamate/glycine are shown in (**D**). Two-way ANOVA showed significant effects of genotype (F_(1,139)_ = 14.282, *p* < 0.001) and treatment (F_(1,139)_ = 156.732, *p* < 0.001) with an interaction between these factors (F_(1,139)_ = 10.484, *p* = 0.002). *p < 0.01, versus AC6^+/+^; ^#^*p* < 0.01 versus AC6^−/−^ neurons without treatment, by Fisher’s LSD post-hoc analysis. The data represent the means ± SEM in each group.

Similar to the findings in the hippocampus of AC6^−/−^ mice (Fig. 4A), we next assessed the levels of CREB, pCREB, and GluN2B in primary hippocampal neurons. Consistent with what were observed in the hippocampal lysates and primary hippocampal neurons of AC6^−/−^ and AC6^+/+^ mice (Figs 1C–H and 4A,B), the amounts of CREB, pCREB, and GluN2B were markedly elevated in AC6^−/−^ hippocampal neurons compared with AC6^+/+^ neurons (Fig. 5A,B, and Supplementary Table 2). In contrast, the protein and transcript level of the AMPA receptor subunit GluA1, insensitive to CREB-mediated gene expression^20^, was not altered in AC6^−/−^ hippocampal neurons (Fig. 5A,B; Supplementary Table 2).

To assess whether the increase in GluN2B protein expression affected signaling triggered by glutamate in primary hippocampal neurons, we determined the increase in the calcium signal triggered by glutamate in primary hippocampal neurons (at DIV14) loaded with Fura2-AM. Consistent with higher levels of GluN2B, glutamate (10 μM) triggered a greater calcium response in AC6^−/−^ neurons than in WT neurons. Treatment with Ro25-6981 (5 μM)^18^ completely removed the difference in the glutamate-triggered calcium signal between AC6^−/−^ and AC6^+/+^ neurons (Fig. 5C,D), indicating that the enhanced GluN2B mediated the increased calcium response to glutamate in AC6^−/−^ neurons. In contrast, calcium responses triggered by high potassium (KCl, 20 mM), which alters membrane potential without stimulating glutamate receptors, were similar in AC6^−/−^ and AC6^+/+^ neurons (Supplementary Fig. 3). Notably, AC6^+/+^ neurons exhibited slower decay of KCl-induced calcium influx in WT neurons when compared with that of AC6^−/−^ neurons, suggesting that AC6 might modulate the reuptake of calcium into intracellular stores (e.g., by the sarcoendoplasmic reticulum calcium transport ATPase, SERCA) and the clearance through plasma membrane Ca^2+^ ATPase in primary neurons. An earlier study reported that AC6 improves calcium uptake in aged hearts^21^. Further investigation into the role of AC6 in controlling the crosstalk between calcium and cAMP in neurons is needed.

### AC6 negatively controls GluN2B-dependent LTD and spatial reversal learning

Alterations in the NMDAR/AMPAR ratio are commonly associated with changes in synaptic plasticity (such as LTP and LTD) and long-term memory^22^,^23^. We next examined whether LTP and LTD were affected in the hippocampus of AC6^−/−^ mice. Electrophysiological analyses revealed that the basal transmission at the Schaffer collateral-CA1 synapse of the hippocampus was similar between adult AC6^−/−^ and AC6^+/+^ mice (Supplementary Fig. 4A). To induce LTP, we first stimulated the hippocampal slices with 2 trains of high-frequency stimulation (HFS, 100 Hz for 1 s, 2 trains at 20 s interval). Surprisingly, no difference in LTP was observed between the hippocampal slices of AC6^−/−^ and AC6^+/+^ mice under the conditions tested (AC6^+/+^, 129.45 ± 2.41% vs. AC6^−/−^, 138.5 ± 5.87%; *p* = 0.22; Supplementary Fig. 4B). Two additional protocols for LTP induction (one train of HFS, 100 Hz for 1 s; one train of theta-burst stimulation (TBS), 5 bursts of 4 pulses at 100 Hz) were also tested. Again, no difference in the LTP induction was observed (Supplementary Fig. 4C,D).

**Figure 6:**
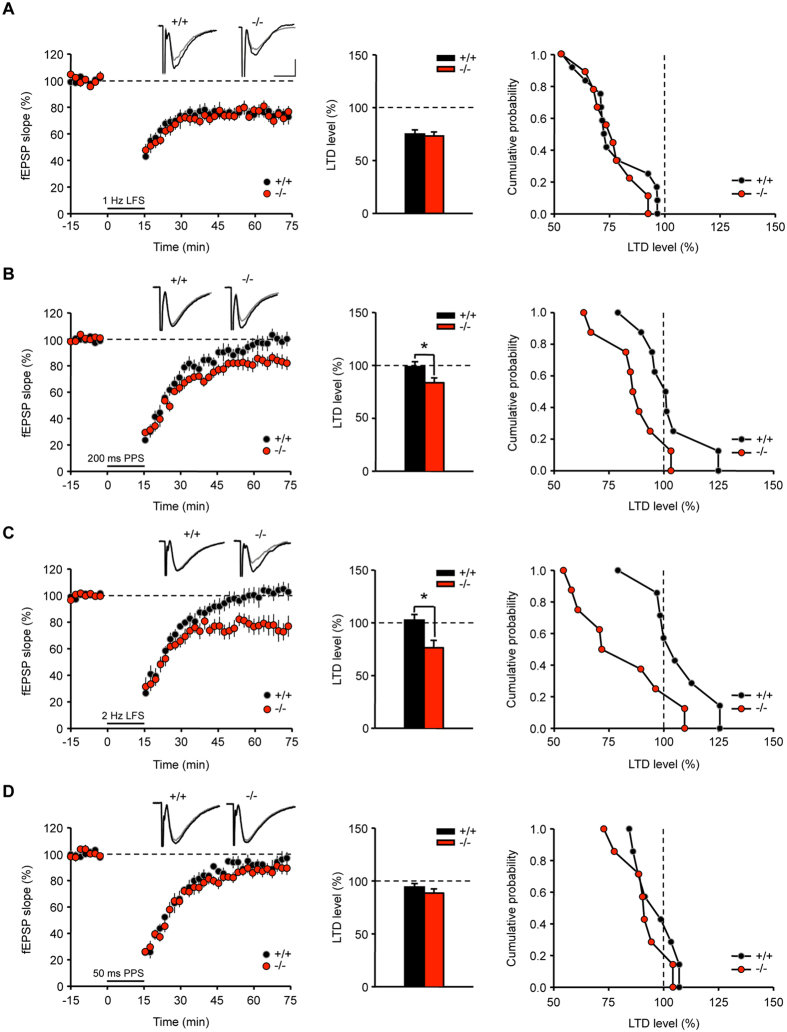
Adult AC6^−/−^ mice showed enhanced NMDAR-dependent LTD at hippocampal Schaffer collateral-CA1 synapses. (**A**) Hippocampal Schaffer collateral-CA1 LTD of AC6^+/+^ (2–3 week-old, n = 12 slice from 3 mice) and AC6^−/−^ mice (2–3 week-old, n = 9 slices from 3 mice) were induced using a single train of LFS (900 pulses at 1 Hz). (**B**,**C**) NMDAR-dependent hippocampal LTD of AC6^+/+^ (3–4-month-old, n = 7–8 slices from 6 mice) and AC6^−/−^ (3–4-month-old, n = 7–8 slices from 5–6 mice) mice were induced by (**B**) paired-pulse stimulation (200 ms PPS; 900 pairs) or (**C**) a single train of low-frequency stimulation (LFS; 1800 pulses at 2 Hz for NMDAR-dependent). (**D**) NMDAR-independent hippocampal LTD of AC6^+/+^ (3–4-month-old, n = 5 slices from 3 mice) and AC6^−/−^ (3–4-month-old, n = 7 slices from 3 mice) mice were induced by paired-pulse stimulation (50 ms PPS; 900 pairs). The representative traces showed the fEPSP of AC6^+/+^ (left) and AC6^−/−^ (right) mice before (black) and 1 hr after (gray) the LTD induction. Scale bars: 0.2 mV, 10 ms. LTD was recorded for 1 hr. The summary and cumulative plots of the LTD levels are shown in the middle and right panels, respectively. The data are reported as the means ± SEM. *p < 0.05, vs. the control littermates, by Wilcoxon rank-sum test.

We next evaluated whether the induction of LTD, another type of long-lasting synaptic plasticity, was altered by the absence of AC6. The classical low-frequency stimulation (LFS, 900 pulses at 1 Hz) protocol to induce LTD was first conducted in young AC6^−/−^ and AC6^+/+^ mice (2–3 weeks old) and showed no significant differences (Fig. 6A).

Compared to young adult mice, LTD is difficult to induce in the adult hippocampus and the protocol mentioned above has been known to be ineffective for LTD induction in adult mice (3–6 months)^24^. Thus, we employed a modified protocol, which has been used to induce hippocampal CA1 LTD in adult rat slices^25^. NMDAR-dependent LTD was induced using a low-frequency paired-pulse stimulation protocol [“200 ms PPS”; 200 ms paired-pulse interval (PPI), 900 pairs]. We observed significant LTD induction in the hippocampal slices of AC6^−/−^ mice, though AC6^+/+^ mice exhibited little LTD (AC6^+/+^, 98.8 ± 4.66% vs. AC6^−/−^, 83.65 ± 4.65%; *p < 0.05, Fig. 6B). The reason that we were not able to induce LTD in the adult AC6^+/+^ mouse hippocampus is unclear. Because the original protocol was designed for rats^25^, species differences may contribute to this failure of LTD induction in AC6^+/+^ mice. Importantly, an evident LTD was induced in AC6^−/−^ mice under the same condition, indicating that AC6^−/−^ mice were more prone to LTD induction than WT mice. Similarly, only AC6^−/−^ hippocampal slices, but not those of AC6^+/+^ controls, exhibited LTD response to another NMDAR-dependent LTD protocol (1800 pulses at 2 Hz, Fig. 6C)^26^. In contrast, NMDAR-independent LTD induced through a single PP-LFS (“50 ms PPS”; 50 ms PPI, 900 pairs)^25^ was similar between AC6^−/−^ and AC6^+/+^ mice (Fig. 6D). Taken together, these data suggest that AC6 negatively regulates synaptic plasticity through the regulation of NMDAR-dependent LTD in the adult hippocampus.

**Figure 7:**
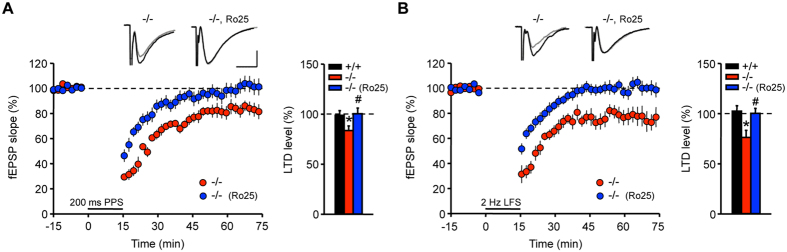
The GluN2B-specific inhibitor Ro25-6981 blocked the enhanced NMDAR-dependent LTD in AC6 KO mice. (**A**,**B**) Hippocampal slices prepared from 3–6-month-old AC6^−/−^ mice were treated with or without Ro25-6981 (Ro25, 5 μM) during the experiment. NMDAR-dependent LTD at Schaffer collateral-CA1 synapses was induced by (**A**) PP-LFS (200 ms PPS) (AC6^+/+^, n = 8 slices from 6 mice, black; AC6^−/−^, n = 8 slices from 5 mice, red; AC6^−/−^ with Ro25, n = 9 slices from 5 mice, blue) or (**B**) LFS (1800 pulses/2 Hz) (AC6^+/+^, n = 7 slices from 6 mice, close circle; AC6^−/−^, n = 8 slices from 7 mice, red; AC6^−/−^ with Ro25, n = 7 slices from 6 mice, blue). The representative traces showed the fEPSP of AC6^+/+^ (left) and AC6^−/−^ (right) mice before (black) and 1 hr after (gray) the LTD induction. Scale bars: 0.2 mV, 10 ms. LTD was recorded for 1 hr. Summary of LTD level is shown in the right panel. The data represent the means ± SEM. The data shown in (**A**,**B**) were analyzed using Wilcoxon rank-sum test. *p < 0.05, vs. the AC6^+/+^ control group. ^#^*p* < 0.05, vs. the AC6^−/−^ group.

Previous studies have shown that GluN2B-containing NMDARs are involved in hippocampal LTD^27^,^28^. During development, GluN2B is downregulated whereas the threshold for LTD induction is increased^29^. To assess whether the increased expression of GluN2B in AC6^−/−^ mice contributes to greater LTD in the hippocampus, we treated hippocampal slices with a GluN2B antagonist (Ro25-6981, 5 μM)^18^ in the LTD experiment. As shown in Fig. 7A, Ro25-6981 eliminated the NMDAR-dependent LTD (200 ms PPS; 200 ms PPI; 900 pairs) in the adult hippocampal slices of AC6^−/−^ mice (100.46 ± 5.61% ^#^*p* < 0.05, vs. AC6^−/−^ neurons without treatment, 83.65 ± 4.65%, Fig. 7A). A similar blocking effect of Ro25-6981 was also observed using an LFS protocol for LTD (2 Hz LFS; 1800 pulses at 2 Hz; 100.26 ± 4.91%, ^#^*p* < 0.05, vs. AC6^−/−^ neurons without treatment, 76.31 ± 7.07%, Fig. 7B).

**Figure 8:**
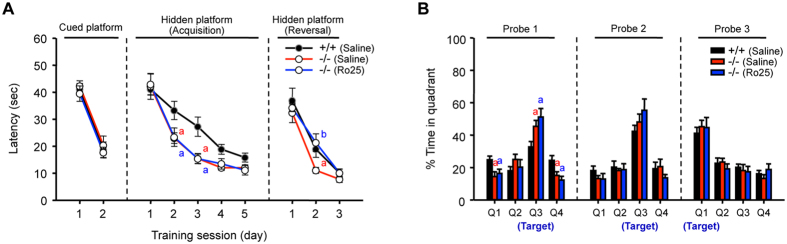
The GluN2B-specific inhibitor Ro25-6981 blocked the enhanced spatial reversal learning in AC6 KO mice. (**A**,**B**) AC6^+/+^ and AC6^−/−^ mice at 3–6 months of age received daily injections with saline or Ro25 (6 mg/kg, i.p.) 1 hour before each training session. (**A**) The escape latency of AC6^+/+^ (Saline, n = 7, close circle) and AC6^−/−^ mice (Saline, n = 6, open circle with red line; Ro25, n = 6, open circle with blue line) to the cued, hidden, and reversal-located hidden platforms was recorded for 2, 5, and 3 days, respectively. (**B**) For probe trials on the training day 3 (probe 1), day 5 (probe 2), and the reversal training day 3 (probe 3), the percentage of time for which each mouse swam in all four quadrants was measured. The quadrant 3 (Q3) is the target quadrant. The data represent the means ± SEM. (**A**) For acquisition, two-way ANOVA showed significant effects of treatment (F_(1,50)_ = 0.035, *p* = 0.853) and training day (F_(4,50)_ = 47.72, *p* < 0.001) with no interaction between these factors (F_(4,50)_ = 0.058, *p* = 0.994). For reversal learning of control groups (saline), two-way ANOVA showed significant effects of genotype (F_(1,33)_ = 5.132, *p* = 0.03) and training day (F_(2,33)_ = 42.301, *p* < 0.001) with no interaction between these factors (F_(2,33)_ = 0.754, *p* = 0.478). For reversal learning of AC6^−/−^ mice with and without Ro25, two-way ANOVA showed significant effects of treatment (F_(1,30)_ = 6.908, *p* = 0.01) and training day (F_(2,30)_ = 62.088, *p* < 0.001) with no interaction between these factors. (**B**) For Probe 1, two-way ANOVA showed interaction between genotype and quadrant (F_(3,88)_ = 13.918, *p* < 0.001) but not between genotype and treatment (F_(1,88)_ = 0.0002, *p* = 0.99). For probe 2 and 3, two-way ANOVA showed no interaction between genotype and quadrant (Probe 2, F_(3,88)_ = 1.687, *p* = 0.176; Probe 3, F_(3,88)_ = 0.631, *p* = 0.597) or between genotype and treatment (Probe 2, F_(1,88)_ = 0.001, *p* = 0.97; Probe 3, F_(1,88)_ = 0.00001, *p* = 0.99). ^a^*p* < 0.05, versus the AC6^+/+^ saline-treated group; ^b^*p* < 0.05, vs. the AC6^−/−^ saline-treated group by Fisher’s LSD post-hoc analysis.

Because LTD is crucial for the acquisition and spatial reversal of memory in the water maze task^30^, we examined whether the enhanced expression of GluN2B in AC6^−/−^ mice contributes to improved learning ability. The mice were treated with Ro25-6981 (6 mg/kg, i.p.)^27^ or vehicle (saline, i.p.) 1 h before the first trial on each training day. Daily injection of Ro25-6981 did not affect the enhanced learning ability of AC6^−/−^ mice in the water maze analysis (Fig. 8A). In the spatial reversal-learning task, AC6^−/−^ mice also showed a higher spatial reversal learning ability than AC6^+/+^ mice (Fig. 8B). Importantly, Ro25-6981 abolished the enhanced spatial reversal learning ability of AC6^−/−^ mice (Fig. 8A) without affecting the memory retention ability shown in the probe tests on spatial learning days 3 (probe 1) and 5 (probe 2) and reversal-learning day 3 (probe 3) (Fig. 8B). Consistent with a previous finding^31^, Ro25-6981 had no effect on swimming speed (Supplementary Fig. 1J), suggesting that Ro25-6981 at the tested dose (6 mg/kg) did not affect the motor function of AC6^+/+^ and AC6^−/−^ mice. Collectively, these data suggest that AC6 negatively regulates reversal learning ability and NMDAR-dependent LTD in the adult hippocampus by modulating the expression of GluN2B.

## Discussion

By using a mouse model lacking AC6^32^, we demonstrated in the present study that AC6 negatively regulates hippocampus-dependent learning without altering amygdala-dependent memory and anxiety-like behaviors (Fig. 3C–F, Supplementary Fig. 1D,E). Biochemical analyses revealed that AC6 is expressed in the postsynaptic neurons of the hippocampus (Supplementary Fig. 5A,C). Genetic removal of AC6 did not affect the expression levels of other calcium-sensitive ACs (Supplementary Fig. 5). The lack of AC6 led to elevated expression of CREB in hippocampal neurons, which can be rescued by exogenous expression of AC6 and a cyclase-dead AC6 variant (AC6_D426A_) but not by an AC6 variant that lacks the regulatory domain (AC6_N-del_, Fig. 2A,B). Enhanced levels of pCREB and CREB in AC6^−/−^ hippocampal neurons subsequently increased the expression of a downstream target of CREB (i.e., GluN2B) as well as the GluN2B-containing NMDAR-mediated calcium response, EPSCs, and LTD in the hippocampus of AC6^−/−^ mice (Figs 1C–H, 4, 5, 6, 7). Consistently, AC6^−/−^ mice also exhibited enhanced GluN2B-dependent reversal learning compared with AC6^+/+^ mice (Fig. 8). Taken together, our findings suggest that AC6 negatively modulates hippocampus-dependent spatial learning via controlling the levels of activated CREB and GluN2B in the hippocampus.

We are particularly interested in the calcium-sensitive ACs (AC1, AC5, AC6, and AC8) because cAMP and calcium are two major intracellular signals that control neuronal functions, and tight regulation between these two signal messages has been widely observed^1^. AC1 and AC8 are two Ca^2+^-stimulated ACs that are expressed in many brain areas (including the hippocampus and cerebral cortex)^33^. In the hippocampus, AC1 and AC8 are expressed in the post-synaptic and pre-synaptic fractions, respectively. The absence of AC1 and AC8 impairs the MAPK cascade in the hippocampus and disrupts spatial memory in mice^2^,^34^,^35^, suggesting that Ca^2+^-stimulated ACs play a critical role in synaptic plasticity. In contrast, the roles of Ca^2+^-inhibited ACs (i.e., AC5 and AC6) in neuronal activity have not been well documented due to the lack of specific antibodies against AC5 and AC6. By using an AC5-specific antibody (Supplementary Fig. 6), we demonstrated that AC5 is expressed in both post- and pre-synaptic fractions of the hippocampus (Supplementary Fig. 5B,C). Conversely, AC6 is primarily detected in post-synaptic fractions (Supplementary Fig. 5A,C), and functions to negatively regulate the CREB expression (Fig. 1).

Similar to AC6^−/−^ mice, mice with exogenous expression of AC1 in the forebrain (known as AC1-Tg mice) show significantly enhanced levels of pCREB and recognition memory. Unlike AC6^−/−^ mice, however, these AC1-Tg mice exhibited enhanced LTP^36^. Earlier studies have also suggested that AC1 and AC6 interact with different A-kinase-anchoring proteins with different functions (Yotiao and AKAP79/150, respectively^37^,^38^). It is very likely that AC1 and AC6 exist in different microdomains of postsynaptic sites in the hippocampus and function differently.

Our data suggest that AC6 might contribute to the regulation of CREB because of an inverse correlation of AC6 with pCREB and CREB expression during development (Fig. 1A,B). In addition, the exogenous expression of AC6 in AC6^−/−^ neurons completely normalized the abnormal levels of pCREB expression (Fig. 2A,B). Interestingly, the catalytic activity of AC6 is not required for the regulation of CREB because an AC6 variant (AC6_D426A_), which has no cyclase activity, also normalized the level of pCREB in a manner similar to wild-type AC6 (Fig. 2A,B). Thus, a cyclase-independent pathway might be involved in the regulation of pCREB by AC6. An intriguing precedent suggesting that AC6 might function via a cyclase-independent and an activating transcription factor-3-dependent pathway has been previously reported^39^. A notable component that might contribute to the cyclase-independent function of AC6 is the cytosolic N-terminal domain because the exogenous expression of AC6_N-del_ did not normalize the enhanced pCREB levels in AC6^−/−^ neurons (Fig. 2A,B). The AC6N is relatively large (160 amino acids) and accounts for the inhibition of AC6 by protein kinase C or Gαi^9^. At least two proteins that interact with the AC6N have been reported (Snapin and AKAP79/150)^9^. Snapin has been implicated in a wide variety of functions, such as neuritogenesis^5^, Ca^2+^ signaling^40^, and the activity of NMDA receptors^41^. AKAP79/150 is a scaffold protein that regulates the functions of many signaling molecules (e.g., AC5, AC6, β-AR and AMPARs) by forming microdomains in the cell through direct protein-protein interactions^42^,^43^. The concept of microdomains is of particular interest because a few functional GPCRs (including β-AR) and their downstream effectors (e.g., AC and phospholipase C) exist not only in the plasma membrane but also in the nuclear envelope membrane^44^. Previous studies have demonstrated that nuclear β-ARs are functionally coupled to ACs and initiate transcription in cardiomyocytes^45^. Our data showed that AC6N mediates the localization of AC6 in the nuclear envelope membrane (Fig. 2C,D), which might contribute to the control of CREB expression in hippocampal neurons. Whether and how the localization of AC6 in the nuclear membrane activates specific signaling pathways that control gene expression, similar to several GPCRs^44^, is currently unknown. Further characterization of the role of AC6 on the nuclear envelope is needed to unravel this mechanism in detail.

A major consequence of the increased pCREB levels in AC6^−/−^ neurons was the enhanced expression of GluN2B that is regulated by CREB activity^11^,^46^. To date, the role of GluN2B and GluN2A in controlling synaptic plasticity remains controversial. Studies using genetically modified mouse models and cultured hippocampal slices have demonstrated that GluN2B is important for LTP, while GluN2A is required for LTP and LTD^8^,^22^,^23^,^47^,^48^. Conversely, studies using intraperitoneal or intracranial injection of antagonists of GluN2B (e.g., Ro25-6981) and GluN2A (e.g., NVP-AAM077) show that GluN2B and GluN2A are required for LTD and LTP, respectively^28^,^49^. In the present study, given that the level of GluN2A was not changed in the hippocampus of AC6^−/−^ mice (Fig. 4), the GluN2A/GluN2B ratio in the hippocampus of AC6^−/−^ mice was lower than that of AC6^+/+^ mice. This altered GluN2A/GluN2B ratio is of great interest because the GluN2A/GluN2B ratio increases from postnatal development to adulthood, and the enhanced GluN2A/GluN2B ratio has been recently demonstrated to constrain LTD and long-term memory^10^,^22^. Therefore, AC6 is likely to contribute to the regulation of NMDAR-mediated synaptic activity by controlling the GluN2A/GluN2B ratio. Unfortunately, blockage of GluN2B could not reverse the enhanced learning ability of AC6^−/−^ mice (Fig. 8A), further studies will be needed to evaluate whether other AC6- or CREB- regulated molecule(s) are involved in the faster spatial learning process of AC6^−/−^ mice. Nonetheless, consistent with the hypothesis that the GluN2A/GluN2B ratio is negatively associated with LTD, AC6^−/−^ mice, which have a reduced GluN2A/GluN2B ratio compared with AC6^+/+^ mice, exhibited GluN2B-dependent LTD and enhanced spatial reversal learning (Figs 7 and 8).

In addition to LTD, synaptic depotentiation is another form of synaptic plasticity that reverses LTP and has been implicated in memory flexibility^50^. Previous studies demonstrate that the loss of both Ca^2+^-stimulated ACs (e.g., AC1 and AC8) impairs LTD, depotentiation, and memory flexibility^51^. Our study showed that removal of AC6 (a Ca^2+^-inhibited AC) induced GluN2B-dependent LTD and enhanced reversal learning (Figs 6, 7, 8). However, depotentiation is GluN2A-dependent^49^ and requires a different phosphatase-dependent pathway^52^. Therefore, it remains unclear whether depotentiation is affected in AC6^−/−^ mice. Together, these findings suggest that calcium-sensitive ACs are likely to contribute to the control of memory flexibility by modulating the balance between LTP and LTD.

Although GluN2B has been implicated in both LTP and LTD, only LTD was affected in AC6^−/−^ mice (Fig. 6B,C). A similar association between the GluN2B-dependent LTD and spatial reversal learning has been reported^53^. In addition, the GluN2B/GluN2A ratio has been shown to affect the induction of LTD but not LTP^22^ (Figs 4 and 6, and Supplementary Fig. 4). Unlike what was observed in mice overexpressing GluN2B (known as GluN2B-Tg mice)^47^, hippocampal LTP in AC6^−/−^ mice was normal (Supplementary Fig. 4). It is interesting to note that GluN2B-Tg mice exhibited at least 4-fold increase in GluN2B expression compared with WT mice, while only a ~25% increase in the level of GluN2B was detected in AC6^−/−^ mice (Fig. 4). We reason that the moderate increase in GluN2B in AC6^−/−^ mice might not be sufficient to alter LTP as was observed in GluN2B-Tg mice.

The localization of NMDAR subunits is critical for their functions. In contrast to synaptic NMDARs that activate ERK1/2, extra-synaptic NMDARs deactivate ERK1/2 and CREB^54^. Therefore, the acute effect of NMDAR activation on CREB activation is highly dependent on the location of NMDARs. In the present study, we demonstrated that the synaptic GluN2B-mediated EPSC was higher in AC6^−/−^ mice (Fig. 4C,D), indicating that the level of synaptic GluN2B in AC6^−/−^ mice was increased compared with AC6^+/+^ mice. Relative to WT mice, the GluN2B-mediated activation of ERK1/2 and CREB might be higher in AC6^−/−^ mice. The activated CREB might bind to the GluN2B promoter to enhance the expression of GluN2B and trigger the feed-forward regulation between GluN2B and CREB in the hippocampus and consequently facilitate neuronal plasticity and the spatial learning process^11^,^46^.

In summary, we have shown that AC6 is an important modulator of synaptic plasticity in the hippocampus. Our results show that AC6 regulates the CREB-mediated cascade in a cyclase-independent and AC6N dependent manner to control the level of GluN2B and LTD in the hippocampus and related reversal learning. Given that the enhanced expression of GluN2B might prevent memory decline during neurodegenerative diseases and aging^55^,^56^, further characterization of the role of AC6 in regulating CREB/GluN2B-dependent memory might provide a novel approach for the development of therapeutic interventions for neurodegenerative diseases.

## Materials and Methods

### Animal care

AC6^−/−^ mice and their littermate controls were maintained in the C57BL/6J (B6) background and genotyped as previously described^32^. The mice were housed individually in ventilated cages (IVC) in an SPF room with a 12/12-hr light-dark cycle and had free access to diet (LabDiet^®^, San Antonio, TX, USA) and water. The accommodation of the mice and all the animal studies were performed in the accordance of the protocols approved by the Institutional Animal Care and Utilization Committee, Academia Sinica, Taiwan.

### Morphological analysis of brain

The brain structures of the mice were measured using micro-magnetic resonance imaging (MRI) analysis in the Taiwan Mouse Clinic and Transgenic Mouse Models Core (TMMC) as previously described^57^. Briefly, the mice were anesthetized using 2 ~ 3% isoflurane at a 1 L/min oxygenation rate. Images of the overall brain structure, hippocampus, and ventricles (right/left lateral ventricle and the 3rd/4th ventricle) were evaluated using a 7 Tesla MRI scanner (Bruker Biospin, Ettlingen, Germany). All images and volumes were processed and measured using ANALYZE (Biomedical Imaging Resource, Mayo Foundation, Rochester, MN, USA). The brain mass (mm^3^) was calculated by subtracting ventricle volume from total brain volume.

### Plasmids

To generate flag-tagged AC6, AC6_D426A_, and AC6_N-del_ expression constructs, the AC6, AC6_D426A_, and AC6_N-del_ fragments were amplified from pcDNA3.1-AC6, pcDNA3.1-AC6_D426A_, and pcDNA3.1-AC6, respectively, that previously generated^5^ by using Phusion High-Fidelity DNA Polymerase (Thermo Scientific, Waltham, MA, USA) and primer sets: (1) For AC6 and AC6 _D426A_: 5′-GGA ATT CAG GAT CCA TGT CAT GGT TTA GCG-3′ and 5′-GGG GTA CCG ATT ACT AGT CTA ACT GCT GGG GCC-3′; (2) For AC6 _N-del_: 5′-GGA ATT CGG CCT TGG GTT TTG ATG ACA CTG-3′ and 5′-GGG GTA CCG ATT ACT AGT CTA ACT GCT GGG GCC-3′, to generate EcoR1 and Kpn1 restriction enzyme cutting sites on the 5′ end and 3′ end, respectively, of amplified AC6 or mutant AC6 fragments. The amplicons were then treated EcoR1 and Kpn1 and subcloned into p3xFLAG-CMV-7.1 vector (Sigma-Aldrich).

### Primary culture, transfection, immunocytochemistry staining, and quantification

Primary hippocampal neurons were isolated from AC6^+/+^ and AC6^−/−^ mice at E18.5 and seeded onto poly-L-lysine-coated glass coverslips. After 10 days *in vitro* (DIV), the cells were transfected with the indicated constructs using Lipofectamine^®^ 2000 (Invitrogen, Carlsbad, CA, USA) as previously described^5^,^14^ with slight modifications, which have been detailed in the Supplementary Experimental Procedures. At DIV14, the cells were fixed and immunostained using the indicated primary antibodies (anti-Flag, 1:500, Sigma-Aldrich, St. Louis, MO, USA; anti-phospho-CREB, 1:1000, Millipore, Bedford, MA, USA; anti-Lamin B1, 1:500, Abcam, Cambridge, UK; anti-KCC2, 1:500, Millipore) following the protocols described in the Supplementary Experimental Procedures. The images were acquired using an LSM780 confocal microscope with ZEN software (Carl Zeiss, Germany). The intensity of pCREB was analyzed using ImageJ (NIH, Bethesda, MD, USA). At least 120 cells in each condition were scored. The relative pCREB intensity was divided by the nuclear area and normalized to that of the control group.

### Calcium imaging

Primary hippocampal neurons were seeded onto poly-L-lysine coated 22 × 22 mm glass coverslips and cultured for 14 days. The cells (DIV14) were loaded with 2 μM Fura-2/AM (Invitrogen) in HBSS buffer (100 mM NaCl, 2 mM KCl, 1 mM CaCl_2_, 1 mM MgCl_2_, 1 mM NaH_2_PO_4_, 4.2 mM NaHCO_3_, 12.5 mM HEPES, and 10 mM glucose at pH 7.4) and incubated at 37 °C for 45 min. The excess Fura-2/AM was washed out 3 times with HBSS and incubated at 37 °C in HBSS for at least 30 min prior to imaging. The images were acquired using a Zeiss Axiovert 200 inverted fluorescence microscope equipped with an open flow chamber (filled with 1 ml HBSS buffer), heated stage, Xenon lamp, and Photomatrics Cool Snap HQ CCD (Photometrics, Tucson, AZ, USA). Fluorescence ratio of calcium images was analyzed using MetaFlour (Molecular Devices, Downington, PA, USA). The stabilization period of the resting cells was recorded for 5 min. Ca^2+^ bound- and Ca^2+^ free- Fura-2 images were acquired every 2 sec after exciting the cells at 340 nm and 380 nm, respectively. The emission wavelength was 510 nm. In general, 5–10 cells were presented and recorded per field. The intracellular concentration of Ca^2+^ was calculated from the 340/380 nm fluorescence ratios at each time point based on a previously described equation^58^.

### Sample preparation, subcellular fractionation, and Western blot analyses

The hippocampi of AC6^+/+^ and AC6^−/−^ mice were collected and homogenized in ice-cold homogenization solution (0.32 M sucrose, 4 mM HEPES, pH 7.4) containing protease and phosphatase inhibitors using glass tissue grinders (Wheaton Scientific, Millville, USA). The homogenized lysates were further fractionated as detailed in the Supplementary Experimental Procedures. The concentration of proteins was determined by using the Pierce BCA Protein Assay Kit (Thermo Scientific). Samples were separated by 10% SDS-PAGE and transferred to polyvinylidene difluoride (PVDF) membranes (Millipore). The PVDF membranes were blocked with 5% BSA in TBST (20 mM Tris, 150 mM NaCl and 0.05% Tween 20) at RT for 1 hr and incubated with the indicated primary antibody as follows: anti-AC1 (1:500, rabbit, Santa Cruz); anti-AC5 (Supplementary Fig. 6A, 1:5000, rabbit^5^); anti-AC6 (Supplementary Fig. 6B, 1:5000, rabbit^5^); anti-phospho-GluN2B (Tyr^1472^, 1:1000, rabbit, Millipore); anti-phospho-GluA1 (Ser^845^, 1:1000, rabbit, Millipore); anti-GluN2B (1:2000, rabbit, Millipore); anti-GluN2A (1:1000, rabbit, Millipore); anti-GluN1 (1:1000, rabbit, Cell Signaling Technology, Danvers, MA, USA); anti-GluA1 (1:1000, rabbit, Millipore); anti-GluA2 (1:1000, mouse, Millipore); anti-phospho-CREB (1:1000, rabbit, Millipore); anti-CREB (1:1000, rabbit, Millipore); anti-PSD95 (1:2000, mouse, Millipore); anti-Syntaxin1 (1:2000, mouse, Santa Cruz); anti-PARP (1:5000, Rb, Santa Cruz); anti-α-tubulin (1:5000, mouse, Sigma-Aldrich); anti-α-actin (1:5000, Rb, Sigma-Aldrich) at 4 °C overnight. After removing the primary antibody(ies), the PVDF membranes were washed with TBST for 4 times (10 min each), followed by incubating the membranes with horseradish peroxidase (HRP)-conjugated secondary antibodies [goat anti- mouse IgG, goat anti-rabbit IgG (Jackson ImmunoResearch, West Grove, PA, USA)] at 1:10000 dilutions at RT for 1 hr. After washes for 4 times (10 min each), immunoreactive bands were visualized by using an enhanced chemiluminescence (ECL) (Perkin-Elmer Life, Boston, MA, USA). Uncropped western blot images were shown in Supplementary Fig. 7. The intensity of immunoreactive band was quantified using ImageJ (NIH). At least 3 independent experiments were performed in each group. The values are presented as the means ± SEM.

### Cognitive function assessment

To examine contextual and cued memory, the mice were placed in the fear-conditioning chamber (Med Associates, Saint Albans, VT, USA) and then received two pairs of conditioned stimuli (CS, white noise, 90 dB, 30 sec)-unconditioned stimuli (US, electric foot shock, 0.5 mA, 2 sec) in a 2-min interval as previously described^57^ and detailed in the Supplementary Experimental Procedures. The freezing behavior of the mice during these tests was recorded and analyzed. Short- and long-term memories were analyzed 1 and 24 hr, respectively, after receiving the CS-US stimulus. To examine spatial and reversal memory, each mouse was subjected to the Morris water maze test as described in the Supplementary Experimental Procedures. Each mouse underwent a daily four-trial session (60 sec for each with a 30 min interval) with different releasing points into the water. For the spatial memory test, each mouse was trained for seven consecutive days (two days with a visible platform and five days with a hidden platform). For the spatial reversal memory test, each mouse was subjected to a new set of training for three additional days (with hidden platform in the opposite quadrant). To test reference memory, probe tests were performed on the third and fifth days of the spatial memory test and the third day of the reversal test. The swimming path, speed, and time spent in different quadrants were analyzed using the TrackMot video tracking system (Singa Technology, Taiwan).

### Electrophysiology

Transverse hippocampal slices (250–350 μm thick) were prepared from mouse brains as described in the Supplementary Experimental Procedures. A monopolar stimulation electrode was placed on the Schaffer collateral pathway and a recording electrode was placed on the stratum radiatum for extracellular recording. LTP was induced by applying two trains of HFS or TBS and recorded for 1 hr. LTD was induced by applying LFS or PPS. All reagents, including the GluN2B antagonist Ro25-6981, were bath applied at least 1 hr before LTP/LTD induction and throughout the entire experiment. To examine NMDAR-mediated EPSCs, the brain slices were perfused with Mg^2+^-free artificial cerebrospinal fluid (ACSF) with 10 μM CNQX, and whole-cell voltage-clamp recordings were obtained from CA1 pyramidal cells (PCs). To determine the NMDAR/AMPAR ratio, whole-cell voltage-clamp recordings were obtained from CA1 PCs. The intracellular patch pipette solution contained (in mM) 121.5 CsMeSO_3_, 0.1 EGTA, 4 MgCl_2_, 13.5 CsCl, 10 HEPES, 5 QX-314 bromide, 2 Na_2_ATP, 10 Na_2_-phosphocreatine, and 0.3 Na_3_GTP, pH 7.3, adjusted with 1 M CsOH (290–300 mOsm). The CA1 PCs were voltage-clamped at −70 mV and +50 mV, respectively. The NMDAR/AMPAR ratio was determined as the ratio of the EPSC amplitude 60 ms after stimulation recorded at +50 mV to the peak amplitude of the EPSCs recorded at −70 mV.

### Statistical analysis

The data are presented as the mean ± SEM. All statistical analyses were performed using SigmaState 3.5. (Systat Software Inc., San Jose, CA, USA). Student’s *t* test or the Wilcoxon rank-sum test was used to compare the difference between two groups. One-way or two-way ANOVA followed by Fisher’s LSD post hoc analysis was used when the differences among multiple groups were compared. Differences were considered statistically significant when *p* < 0.05.

## Additional Information

**How to cite this article**: Chang, C.-P. *et al*. Type VI adenylyl cyclase negatively regulates GluN2B-mediated LTD and spatial reversal learning. *Sci. Rep.* **6**, 22529; doi: 10.1038/srep22529 (2016).

## Supplementary Material

Supplementary Information
