# Supplementary material for: Type VI adenylyl cyclase negatively regulates GluN2B-mediated LTD and spatial reversal learning
Source: Sci Rep. 2016 Mar 2;6:22529. doi: 10.1038/srep22529 (PMC4773765; doi:10.1038/srep22529)
Supplement: Supplementary Information [file srep22529-s1.pdf]

Type VI adenylyl cyclase negatively regulates GluN2B-mediated LTD and spatial reversal learning

Ching-Pang Chang[1, 2, 4], Cheng-Ta Lee[5], Wen-Hsien Hou[5], Meng-Syuan Lin[1, 2, 4], Hsing-Lin Lai[2], Chen-Li Chien[2], Chen Chang[1,2], Pei-Lin Cheng[3], Cheng-Chang Lien[1, 5, *], and Yijuang Chern[1, 2, 5, *]

(*, *co-corresponding authors.*)

[1] Taiwan International Graduate Program in Molecular Medicine, National Yang-Ming University and Academia Sinica, Taipei, Taiwan; [2] Institute of Biomedical Sciences and [3] Institute of Molecular Biology, Academia Sinica, Taipei, Taiwan; [4] Institute of Biochemistry and Molecular Biology and [5] Institute of Neuroscience, National Yang-Ming University, Taipei, Taiwan.

Inventory of Supplemental Information-

a) Supplemental Figures and Legends

b) Supplemental Tables

c) Supplemental Experimental Procedures

d) Supplemental Experimental Procedures References

Supplementary Figures

Supplementary Figure 1

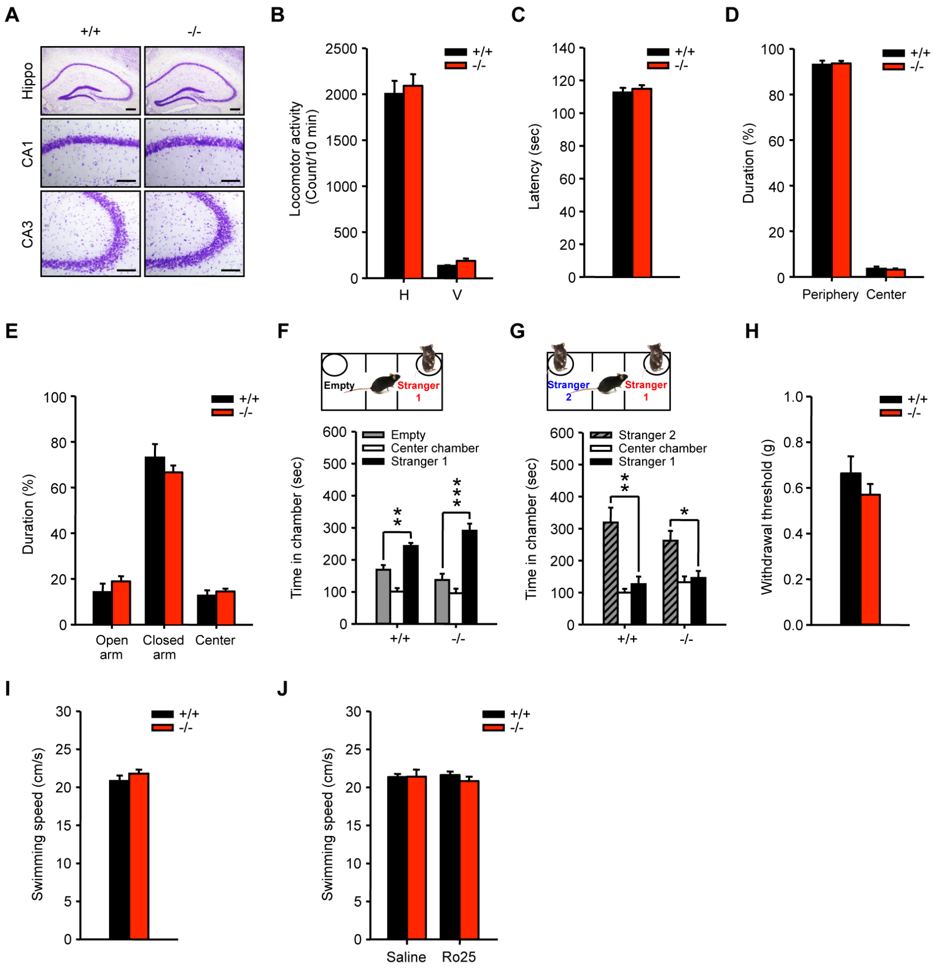

Supplementary Figure 1. AC6$^{-/-}$ mice exhibited normal motor function, anxiety and social behavior, and gross anatomy of the hippocampus

Mice at 3-6 months of age were used for hippocampus morphology and general behavior analysis. (**A**) Hippocampal sections (20 μm) from AC6$^{+/+}$ (n=3) and AC6$^{-/-}$ (n=3) mice of 3-6 months were used for Nissl staining to examine the morphology of the hippocampus. From top to bottom, the images represented the hippocampus, CA1, and CA3; scale bar, 100 μm. (**B**) Horizontal (H) and vertical (V) locomotor activity of AC6$^{+/+}$ (n=5, black) and AC6$^{-/-}$ (n=5) mice in the activity chamber was measured for 10 min. (**C**) Rotarod performances (fixed speed at 28 rpm) of AC6$^{+/+}$ (n=10, black) and AC6$^{-/-}$ (n=10) mice were evaluated after calculating the time spent on the rod in 2 minutes. (**D, E**) The anxiety-like behavior was tested using open field (**D**) and elevated plus maze tests (**E**). Histograms showing the percent of time spent by AC6$^{+/+}$

(n=6, black) and AC6$^{-/-}$ (n=8) mice in different areas (center and periphery for open field test; open arm, closed arm, and center for elevated plus maze). (**F, G**) Social behavior was analyzed using the three-chamber sociability test. Social affiliation (**F**) and social preference (**G**) were measured by monitoring the time spent in the chamber with stranger 1 (black) and stranger 2 (striped black), respectively. The gray and white bars represent the empty and center chambers, respectively. (**H**) The pain sensation (withdrawal threshold, g) of AC6$^{+/+}$ (n=6) and AC6$^{-/-}$ (n=6) mice was measured by von Frey assay. (I-J) Swimming velocity of non-treated mice (I, AC6$^{+/+}$, n=9; AC6$^{-/-}$, n=7), and treated with saline (J, saline; AC6$^{+/+}$, n=7; AC6$^{-/-}$, n=6) or Ro25 (6 mg/kg, AC6$^{+/+}$, n=7; AC6$^{-/-}$, n=6). The data were presented as the means ± SEM for each group. *$p<0.05$; **$p<0.01$; ***$p<0.001$, by Student's t-test

Supplementary Figure 2

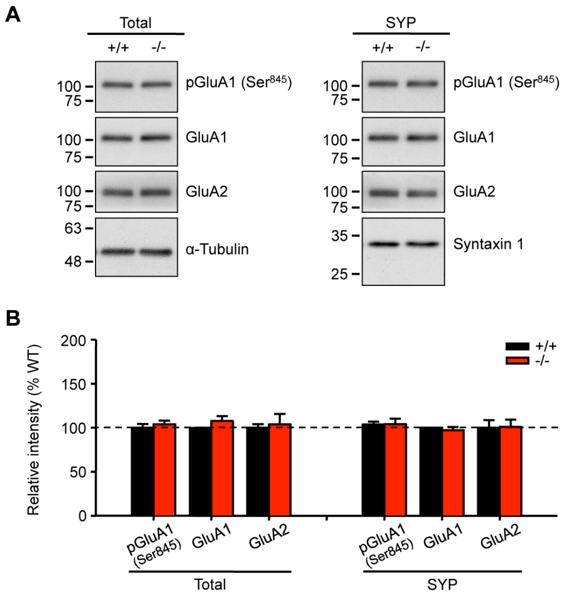

Supplementary Figure 2. AMPAR subunit expression levels were not altered in the hippocampus of AC6$^{-/-}$ mice

(**A-C**) Total hippocampal lysate (Total, 20 µg) and synaptosome fraction proteins (SYP, 5 µg) of AC6$^{+/+}$ (n=3-5) and AC6$^{-/-}$ (n=3-5) 3-6-month-old mice were subjected to WB analysis. (**A**) The expression levels of pGluA1 (Ser^{845}), GluA1, and GluA2 were measured using the indicated antibodies. α-Tubulin and syntaxin 1 were used as internal loading controls for the total lysate and synaptosome fractions, respectively. The protein expression level was quantified and shown in (**B**). The data represent the means ± SEM of three independent experiments. Statistical analyses were conducted using Student's *t*-test.

Supplementary Figure 3

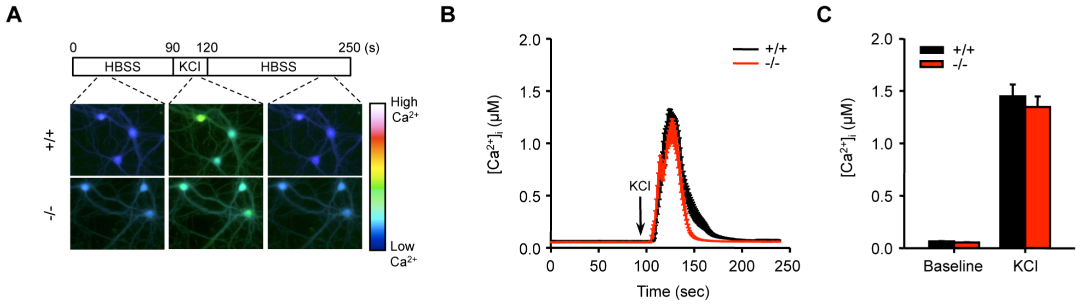

Supplementary Figure 3. AC6$^{-/-}$ primary hippocampal neurons showed normal KCl-evoked calcium responses

Cytoplasmic calcium activity was evaluated in fura-2-loaded neurons. (**A**) Fura-2 ratio (340/380 nm) for the images of DIV14 primary hippocampal AC6$^{+/+}$ (upper) and AC6$^{-/-}$ (lower) neurons before, during, and after KCl treatment. (**B**) KCl (20 mM)-evoked intracellular calcium concentration ($[Ca^{2+}]_i$) response in the DIV14 primary hippocampal neurons of AC6$^{+/+}$ mice (n=21) and AC6$^{-/-}$ mice (n=19). The basal and maximum cytosolic Ca^{2+} responses evoked through KCl are shown in (**C**). The data were presented as the means ± SEM for each group. **p*<0.05, Student's t-test.

Supplementary Figure 4

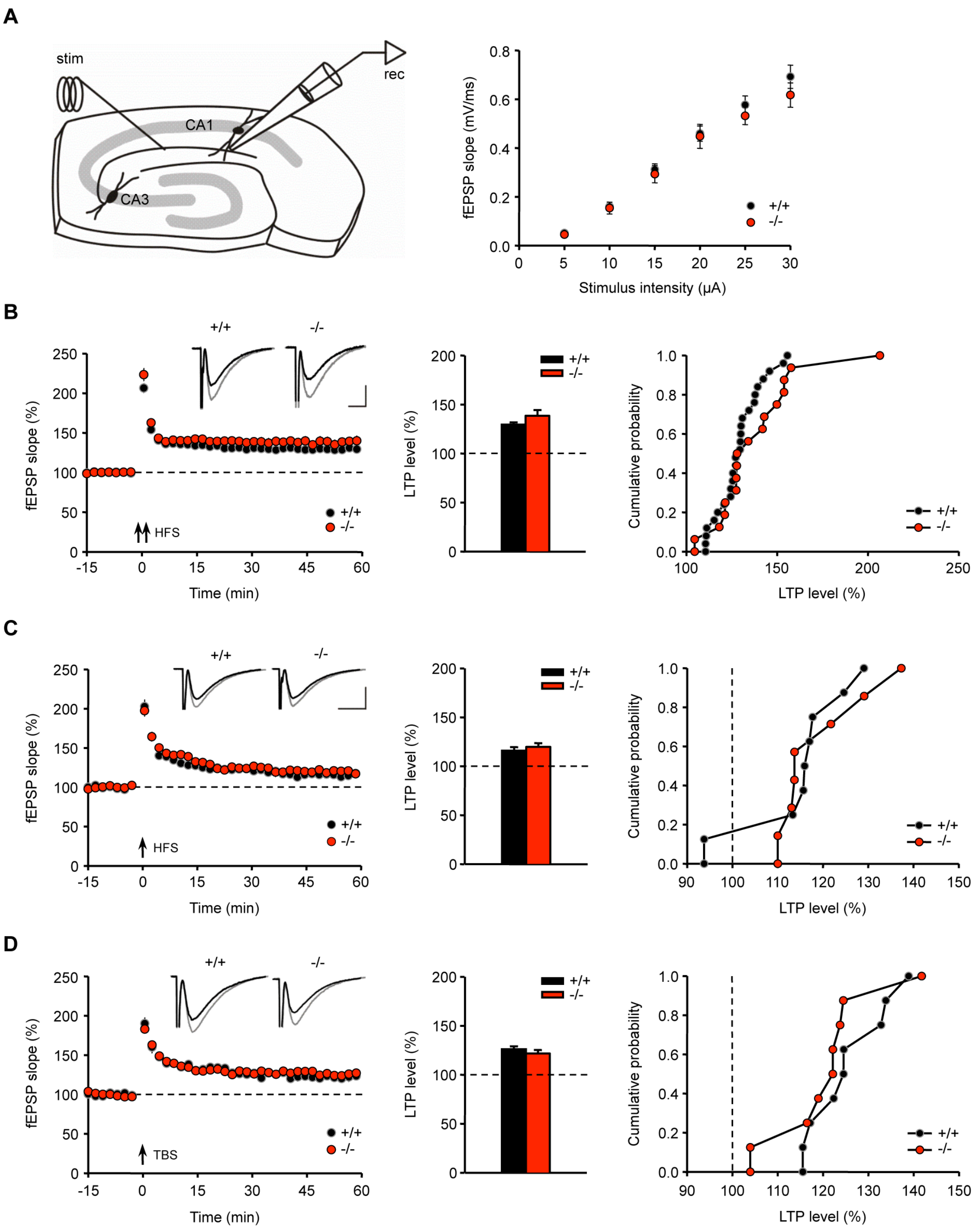

Supplementary Figure 4. There were no differences in the weak stimulation-elicited LTP between AC6$^{-/-}$ and AC6$^{+/+}$ mice

Hippocampal slices for LTP induction were prepared from 3-6-month-old mice. (**A**) Recording configuration (left panel): a stimulation electrode (stim) was placed in the stratum radiatum to activate the Schaffer collaterals; rec represents fEPSP recording. Hippocampal Schaffer collateral-CA1 input/output curves (right panel) from AC6$^{+/+}$

and AC6$^{-/-}$ mice were recorded prior to LTP induction. (**B-D**) Hippocampal Schaffer collateral-CA1 LTP of AC6$^{+/+}$ (n=8-25 slices from 4-13 mice) and AC6$^{-/-}$ mice (n=7-16 slices from 4-10 mice) was induced using (**B**) 2 trains of high-frequency stimulation (HFS; each train includes 100 Hz for 1 s, the train-to-train interval is 20 s), a single train of (**C**) HFS (100 Hz for 1 s) or (**D**) TBS (theta-burst stimulation, 5 bursts of 4 pulses at 100 Hz) and LTP was recorded for 1 h. The representative traces showed the fEPSP of AC6$^{+/+}$ (left) and AC6$^{-/-}$ (right) mice before (black) and 1hr after (gray) the LTP induction. Scale bars: 0.2 mV, 10 ms. The summary and cumulative plot of LTP level are shown in the middle and right panels, respectively. The data were analyzed using Wilcoxon signed-rank test and presented as the means ± SEM for each group

Supplementary Figure 5

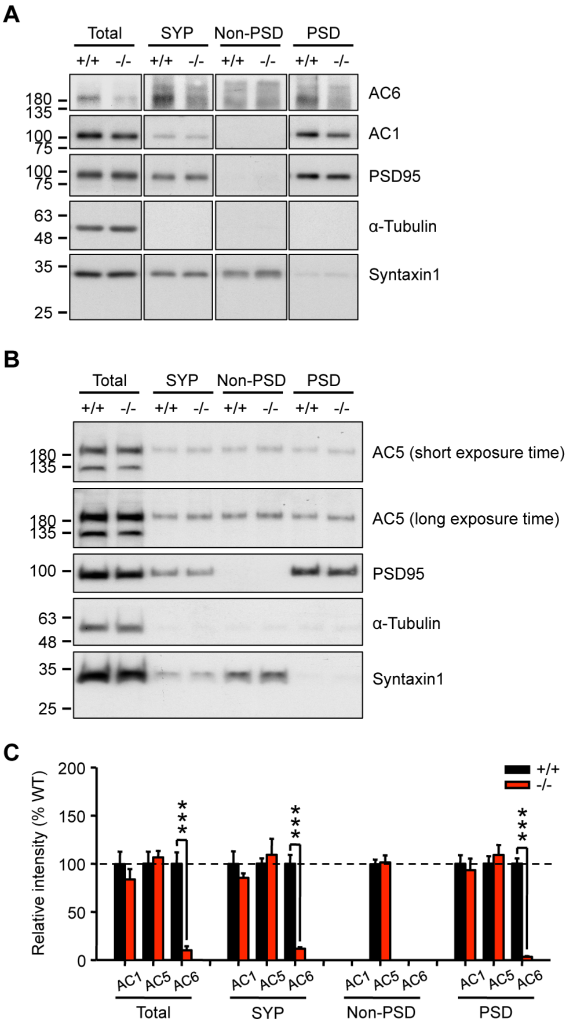

Supplementary Figure 5. AC6 was enriched on postsynaptic membrane in the hippocampus

Total lysate (Total, 20 μg), synaptosome (SYP, 5 μg), non-postsynaptic density (non-PSD, 5 μg), and PSD-enriched fraction proteins (PSD, 5 μg) harvested from AC6$^{+/+}$ (n=3) and AC6$^{-/-}$ (n=3) mice of 3-6 month-old were subjected to WB analysis. The subcellular localization of (**A**) AC1 and AC6, and (**B**) AC5 were detected using the indicated antibody. α-Tubulin, syntaxin 1, and PSD95 were used as the internal loading controls for the total, SYP/non-PSD, and PSD fractions, respectively. The protein expression level was quantified and shown in (**C**). The data represent the means ± SEM of three independent experiments. Statistical analyses were conducted using Student's *t*-test. ***p<0.001, versus control group.

Supplementary Figure 6

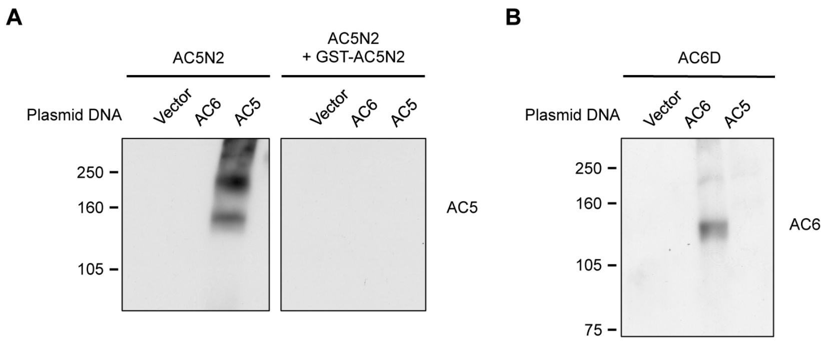

Supplementary Figure 6. Determination of the antibody specificity of AC5N2 and AC6D

Membrane protein (20 μg), harvested from HEK293T cells transfected with pcDNA3-vector, pcDNA3-AC6, or pcDNA3-AC5 as indicated, were subjected to WB analysis and probed with (A) AC5N2 antibody (left panel) or AC5N2 antibody absorbed with GST-AC5N2[1] (100 μg/ml) (right panel), or (B) AC6D[2] to validate the specificity of the indicated antibody.

Supplementary Figure 7

A. Uncropped Fig. 1A

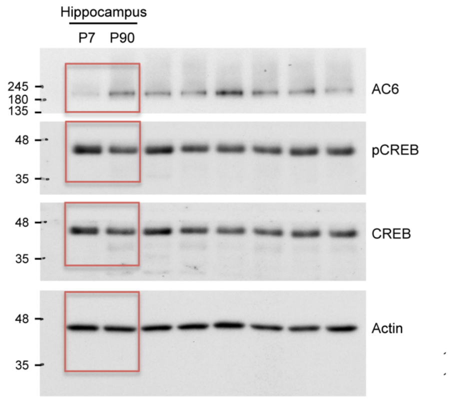

B. Uncropped Fig. 1C

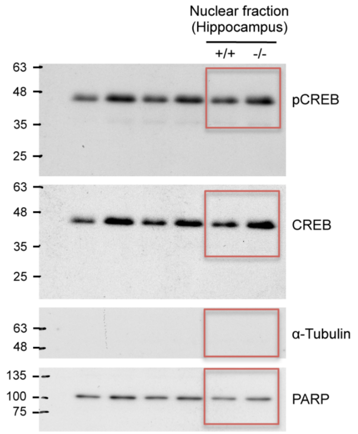

C. Uncropped Fig. 4A

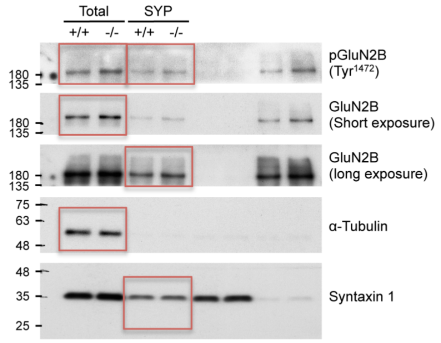

D. Uncropped Fig. 4A

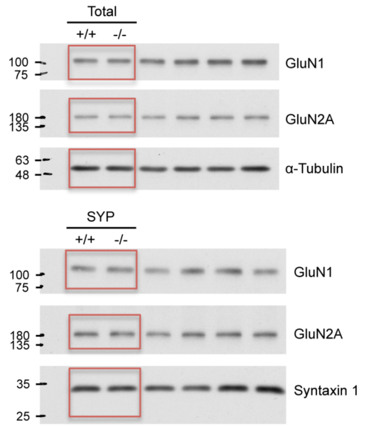

E. Uncropped Fig. 5A

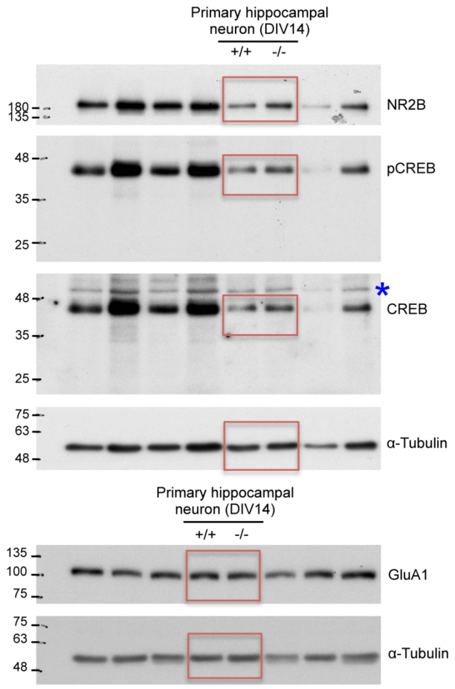

F. Uncropped Fig. S2A

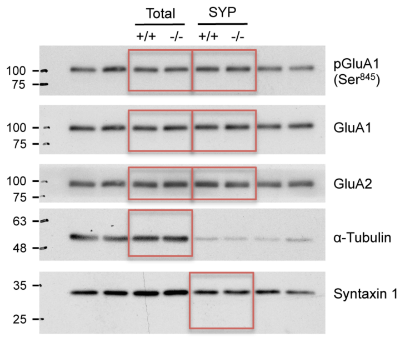

Supplementary Figure 7

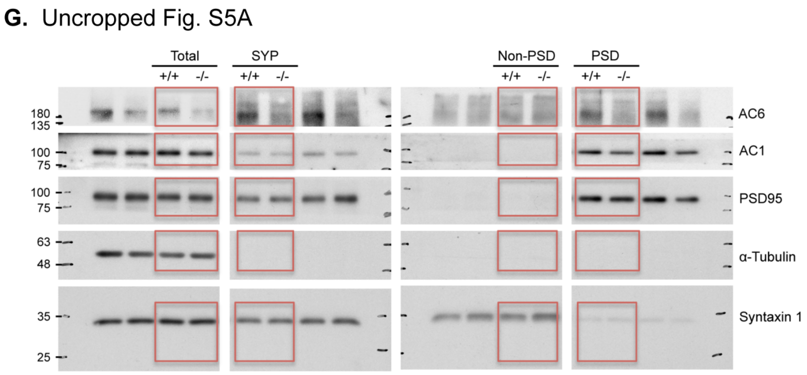

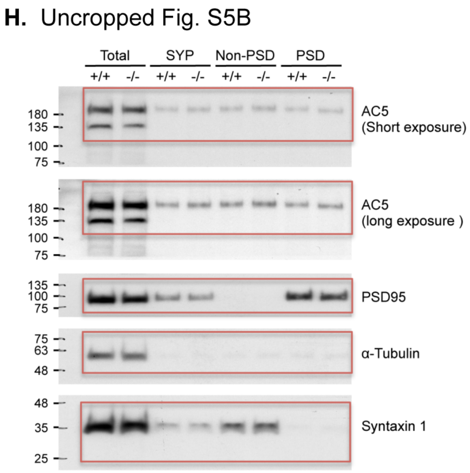

Supplementary Figure 7. Uncropped images of western blots.

Uncropped western blot images used in (A) Fig. 1A, (B) Fig. 1C, (C and D) Fig 4A, (E) Fig. 5A, non-specific bands were indicated with blue asterisk, (F) Supplementary Fig. 2A, (G) Supplementary Fig. 5A, and (H) Supplementary Fig. 5B are shown. The region of the western blots shown in the corresponding figures are marked by red brackets. The blue star indicates a non-specific band.

Supplemental Tables

Table S1. Related to Figure 4, the gene expression levels of NMDAR subunit, GluN2B and CREB were increased in the AC6$^{-/-}$ hippocampus without affecting AMPAR subunit, GluA1.

Gene	Gene name	Adult hippocampus<br>Relative mRNA expression (%)<br>+/+<br>(n=6)	 <br>-/-<br>(n=6)
**GluN2B**	Grin2b, glutamate receptor, ionotropic, NMDA2B (epsilon 2)	101.8 ± 4.2	122.2 ± 5.3*
**GluA1**	Gria1, glutamate receptor, ionotropic, AMPA1 (alpha 1)	100.6 ± 2.7	107.8 ± 3.4
**CREB**	Creb1, cAMP responsive element binding protein 1	100.9 ± 3.1	121.9 ± 5.3**

Expression level of each gene was normalized with average value of AC6$^{+/+}$ control. Values were presented as the mean ± SEM and analyzed with Student's t-test. *p<0.05; **p<0.01, statistically significant versus the control group.

Table S2. Related to Figure 5, the gene expression levels of NMDAR subunit, GluN2B and CREB were increased in the AC6$^{-/-}$ primary hippocampal neurons without affecting AMPAR subunit, GluA1.

		Primary Hippocampal neurons Relative mRNA expression (%)	
		+/+	-/-
**Gene**	**Gene name**	**(DIV14, n=5)**	**(DIV14, n=5)**
**GluN2B**	Grin2b, glutamate receptor, ionotropic, NMDA2B (epsilon 2)	100.1 ± 2.7	289.1 ± 28.5***
**GluA1**	Gria1, glutamate receptor, ionotropic, AMPA1 (alpha 1)	100.1 ± 2.3	95.8 ± 9.9
**CREB**	Creb1, cAMP responsive element binding protein 1	100.3 ± 3.7	170.7 ± 4.0**

Expression level of each gene was normalized with average value of AC6$^{+/+}$ control. Values were presented as the mean ± SEM and analyzed with Student's t-test. ***p*<0.01; ****p*<0.001, statistically significant versus the control group.

Table S3. Related to Supplemental Experimental Procedures, Primer sets for Quantitative PCR

Gene name	Primer sequences	
GluN2B<br>(Grin2b, NM_008171)	Forward<br>Reverse	5'-CATCCGAAGCTGGTGATAATCC-3'<br>5'-GGTGCCTCCTCCAAGGTAACGA-3'
GluA1<br>(Gria1, NM_001113325)	Forward<br>Reverse	5'-TCAGCGACGGCAAATACGGAG-3'<br>5'-CCAAGGTTATGGTCAAGGGGG-3'
CREB<br>(Creb1, NM_133828)	Forward<br>Reverse	5'-GAGACTTCAGCCCCTGCCATCAC-3'<br>5'-CCCCATCCGTACCATTGTTAGCC-3'
GAPDH<br>(Gapdh, NM_008084)	Forward<br>Reverse	5'-TGACATCAAGAAGGTGGTGAAG-3'<br>5'-AGAGTGGGAGTTGCTGTTGAAG-3'

Supplemental Experimental Procedures

Brain tissue preparation and Nissl staining

The mice were initially anesthetized with 80 mg/kg sodium pentobarbital (i.p.) and subsequently perfused intracardially with 0.9% NaCl and 4% paraformaldehyde (PFA) in 0.1 M phosphate buffer (PB, pH 7.4). The brains were then collected and postfixed in 4% PFA for 24 hr, followed by incubation in 30% sucrose solution in 0.1 M PB for 48 hr. The brains were frozen on dry ice and subjected to coronal sectioning (20 μm thickness) using a freezing sliding microtome (HM430, Microm, Germany). The hippocampal sections were then stained with Nissl solution (0.1% cresyl violet, Sigma-Aldrich, St. Louis, MO, USA) for 20 min at room temperature (RT) and washed with distilled water, followed by a standard dehydrating procedure (85% EtOH → 95% EtOH → 100% EtOH → Xylene). The stained hippocampal sections were mounted with permanent mounting solution (Malinol; Mutoh Chemical Co., Tokyo, Japan). The images were acquired using a system microscope (BX51, Olympus Optical Co., Tokyo, Japan) equipped with a microscope digital camera system (DP70, Olympus Optical Co).

Cell culture and transfection

HEK-293T cells were cultured as previous described[1]. For primary hippocampal neurons, neurons were isolated from $AC6^{+/+}$ and $AC6^{-/-}$ mouse embryos at E18.5. The isolated hippocampi were first washed with ice-cold Hanks' balanced salt solution (HBSS) and then incubated with 0.5 mg/ml trypsin-EDTA (Thermo Scientific) at 37°C for 10 min. Fetal bovine serum (FBS) was added to neutralize the effect of trypsin-EDTA. The trypsin-containing buffer was carefully removed, and the hippocampi were resuspended in Neural Culture Medium 1 [Modified Eagle's medium (MEM) containing 1% insulin-transferrin-selenium (ITS) supplement, 5% FBS, 5% horse serum, 0.6% glucose, 0.5 mM L-glutamine, 1% penicillin-Streptomycin (PS)]. The neurons were seeded onto 0.1 mg/ml poly-L-lysine coated 22x22 mm rectangular glass coverslips or 12-mm round glass coverslips in 6-well plates at a density of 1-1.5x10^5 cells/ml and incubated at 37°C with 5% CO_2. After one hour, the Neural Culture Medium 1 was replaced with Neural Culture Medium 2 (Neurobasal medium containing 2% B27 supplement, 0.5 mM L-glutamine, 12.5 μM L-glutamate). At 3 days-*in-vitro* (DIV), 50% of the medium was replaced with Neural Culture Medium 2 (with 2% B27 supplement, 0.5 mM L-glutamine) plus 1.25 μM cytosine arabinoside (AraC) to remove dividing cells, such as astrocytes and

fibroblasts. Half of the Neural Culture Medium 2 was replaced with the same volume of the freshly prepared medium at DIV10. HEK-293T cells and primary hippocampal neurons at DIV10 were transfected with the indicated constructs using Lipofectamine 2000 (Invitrogen, Carlsbad, CA, USA) according to the manufacturer's instructions, with slight modifications.

Immunocytochemistry staining

Primary hippocampal neurons (DIV14) transfected with 3xFlag-AC6, 3xFlag-$AC6_{D246A}$, 3xFlag-$AC6_{N\text{-}del}$, or 3xFlag-vector at DIV10 were fixed in 4% paraformaldehyde/4% sucrose in PBS at RT for 30 min. The neurons were permeabilized with 0.05% NP40 in 0.1 M PBS for 10 min, followed by incubation with the blocking solution (2% BSA and 2% normal goat serum in 0.1 M PBS) at RT for 1 hour. After additional washes with 0.1 M PBS, the neurons were incubated with the indicated primary antibody: anti-Flag (1:500, mouse, Sigma-Aldrich), anti-phospho-CREB (1:1000, rabbit, Millipore, Bedford, MA, USA), anti-MAP2 (1:1000, mouse, Millipore), anti-Lamin B1 (1:500, rabbit, Abcam, Cambridge, UK), and anti-KCC2 (1:500, rabbit, Millipore) in 0.1 M PBS (containing 1% NGS) at 4°C overnight. The neurons were subsequently washed with 0.1 M PBS and incubated with the corresponding fluorescent-conjugated secondary antibody as follows: Alexa Fluor® 488 goat anti-mouse IgG (1:250, Life Technologies, Carlsbad, CA, USA) and Alexa Fluor® 568 goat anti-rabbit IgG (1:250, Life Technologies) in 0.1 M PBS plus 1% NGS at RT for 2 hr, followed by the counterstaining of nuclei with Hoechst 33258 (1:10000, Life Technologies). After additional washes with 0.1 M PBS, the neurons were mounted with VECTASHIELD® mounting media (Vector Laboratories, Inc., CA, USA). Fluorescence images were acquired using an LSM780 confocal microscope with ZEN software (Carl Zeiss, Germany), and analyzed using ImageJ software (NIH, Bethesda, MD, USA).

Subcellular fractionation

Subcellular fractions of $AC6^{+/+}$ and $AC6^{-/-}$ mouse brains were obtained using biochemical fractionation techniques as previously described[3], with slight modifications. All reagents/solutions (including H_2O) contained protease inhibitors (cOmplete EDTA-free protease inhibitor cocktail, Roche Diagnostics, Mannheim, Germany) and phosphatase inhibitors (PhosSTOP inhibitor cocktails, Roche Diagnostics). Briefly, the hippocampus was first homogenized in ice-cold

homogenization solution 1 (0.32 M sucrose, 4 mM HEPES, pH 7.4). Part of the hippocampus lysate was saved as the total lysate. The supernatant (S1) was collected after centrifuging the remaining hippocampus lysate at 800 x g at 4°C for 10 min. The pellet (P1) was resuspended in ice-cold homogenization solution 1 and centrifuged at 700 x g at 4°C for 10 min to collect the supernatant (S1'). The cytosolic fraction (S2) was obtained from centrifuging the combined supernatant (S1+S1') at 10,000 x g at 4°C for 15 min. The pellet (P2) was resuspended in ice-cold homogenization solution 1, followed by centrifugation at 10,000 g at 4°C for 15 min. The pellet (P2') was saved as the crude synaptosomal fraction. The remaining P2' fraction was lysed through hypo-osmotic shock in ice-cold H_2O. The solution was rapidly adjusted to 4 mM HEPES by adding 1 M HEPES (pH 7.4) and mixed well in cold room (4°C) for 30 min. After centrifuging at 25,000 x g at 4°C for 20 min, the supernatant was discarded, and the pellet (P3, lysed synaptosomal membrane fraction) was resuspended in ice-cold homogenization solution 1 and subsequently layered on top of a discontinuous sucrose gradient (0.8 M, 1.0 M, 1.2 M sucrose), followed by centrifugation at 30,000 rpm in SW41 Ti at 4°C for 2 hr in a swinging bucket rotor (Beckman Instruments, Fullerton, CA, USA). The synaptic membrane was collected from the layer between 1.0 M and 1.2 M sucrose and diluted to 0.32 M sucrose. The samples were centrifuged at 150,000 x g at 4°C for 30 min in TL100 rotor (Beckman Instruments) to collect the pellets (Syn, synaptosome fraction). For extracting the postsynaptic densities (PSD) and non-PSD fraction, the Syn fraction was resuspended in the ice-cold homogenization solution 2 (2 mM EDTA, pH 8.0; 50 mM HEPES, pH 7.4) containing 0.5% Triton X-100. The sample was rotated in cold room for 15 min, followed by centrifugation at 32,000 x g at 4°C for 20 min in TL100 rotor (Beckman Instruments). The supernatant and pellet were collected as the non-PSD fraction and the PSD fraction, respectively.

RNA extraction and RT-qPCR

RNA extraction was performed following the manufacture's protocol (Life Technologies). The hippocampus tissue (in glass Douncer grinder) or primary hippocampal neurons (in culture dish) were homogenized by using 1 ml TRIzol (Life Technologies). The homogeneous lysate was transferred to RNase-free microfuge tube and incubated at RT for 5 minutes. 0.2 ml Chloroform/Isoamyl was then added into the homogeneous lysate and mixed well by inverting the tube at RT for 3 minutes followed by centrifuged at 12,000 X g for 10 minutes. The supernatant was then

transferred into a fresh RNase-free tube and added 0.5 ml isopropyl alcohol to precipitate RNA. After 10 minutes centrifugation at 12,000 X g, the supernatant was discarded the supernatant and pellet was washed twice with 0.5 ml 70% ethanol. The pellet was air-dried for 10 minutes and re-suspendeded in RNase-free distilled water. The nucleic acid isolated was treated with DNase1 (Life Technologies) and RNA was precipitated following the manufacturer's protocol to remove genomic DNA contamination. The total RNA concentration and quality were measured by using NanoDrop spectrophotometer (ND-1000, Thermo Scientific). For complementary DNA (cDNA) synthesis, the total RNA was mixed with 10 mM dNTP and random primers followed by heating the mixture at 65 °C for 5 minutes and incubating on ice for at least 1 minute. After brief centrifugation, the 5X first-strand buffer, 0.1M DTT, RNaseOUT, and SuperScript III reverse transcriptase (Life Sciences) were added into RNA-containing mixture. The tube was then incubated at following temperatures: 25 °C for 5 minutes, 50 °C for 60 minutes, and 70 °C for 15 minutes. For relative quantitation of gene expression level, the synthesized cDNA (20 ng) and SYBR® Green reagent (Life Technologies) were used for qPCR and performed on an ABI PRISM® 7700 Sequence Detection System (Life Technologies). The primer sequences of target genes were shown in Table S3.

Motor function analyses

The motor functions of $AC6^{+/+}$ and $AC6^{-/-}$ mice were examined using the rotarod performance and locomotor activity tests. In rotarod performance, the motor coordination ability of each mouse was assessed using the rotarod apparatus (Ugo Basile, Comerio, Italy). Briefly, the average latency to fall from the rod (28 rpm) of each mouse was recorded for 2 min. Locomotor activity was recorded and analyzed in an activity chamber (Coulbourn Instruments, Allentown, PA, USA) equipped with 16 x 16 infrared sensors for 10 minutes. The horizontal and vertical exploration, which represent locomotion and rearing, respectively, of each mouse was recorded.

von Frey assay

One hour before the experiment, each animal (non-anesthetized) was placed in a small acrylic box (10 cm L x 6 cm W x 10.5 cm H) on wired mesh (65 cm L x 21 cm W x 31 cm H) for habituation. The withdrawal response was measured by poking their hind paw with the Electronic von Frey Anesthesiometer (Model 2392; IITC Life Science, California, USA) equipped with a special Rigid Tip (Rigid Tip+ 0.01 inches

tungsten electrode) with increasing force a total of 5 times (each hindpaw) with a 3 min interval between tests. Data collected from left and right hindpaws were averaged together. The lowest force to evoke a withdrawal response is considered the pain threshold for the tested animal.

Anxiety-related behavior

The anxiety responses of $AC6^{+/+}$ and $AC6^{-/-}$ were assessed using an open field test and elevated plus maze (EPM) as previously described[4,5] with slight modifications. For habituation, the mouse was moved to the experimental room 2 hr before testing. In the EPM test, the mice were placed at the center of EPM with two open arms (30 cm L x 5 cm W) and two closed arms (30 cm L x 5 cm W x 15 cm H). Each mouse was allowed to explore and was recorded for 5 min. The duration of time spent in each arm was analyzed. For the open field test, the mice were placed at the center of the square open-field box (48 cm L x 48 cm W x 35 cm H), followed by recording for 10 min. The distance of each mouse crossing over the center zone (16 cm x 16 cm) and peripheral zone were analyzed. Approximately 80% of the mouse body crossing over arena was defined as the entry of the different arena. All videos were analyzed using Top Scan software (Clever Sys. Inc., Reston, VA, USA).

Three-chamber sociability analyses

Sociability tests were performed as previously described[6], with slight modifications. For habituation, the mice were first placed into the middle compartment (12 cm x 25.4 cm) of a three-compartment chamber (52 cm L x 25.4 cm W x 23 cm H), isolating the isolated the right and left compartments (20 cm x 25.4 cm) with empty wire containment cups (12 cm diameter) in the middle using the removable walls for 5 min. For social affiliation analyses, one mouse (stranger 1) was placed inside a wire containment cup located in one compartment (left or right) of the three-compartment chamber, followed by removing the walls between the compartments. The mouse was allowed to access and explore each compartment for 10 min. For the social preference analysis, the three-compartment chamber was isolated and a second mouse (stranger 2) was placed inside the opposite compartment (right or left), and the walls between the compartments were removed. The exploring activity was recorded for 10 min. The duration spent by each mouse in each compartment was analyzed using Top Scan software (Clever Sys. Inc.).

Fear-conditioning

A fear conditioning chamber (Med Associates, Saint Albans, VT, USA) equipped with auditory cue and electric shock device was used for the fear conditioning test. Briefly, the mice were placed and habituated in the conditioning chamber for 2 min. The mice received a 30-second neutral conditioned stimulus (CS, white noise, 90 dB), followed by a 2-second noxious unconditioned stimulus (US, electric foot shock, 0.5 mA). After an inter-trial interval (2 min), mice received a second identical set of stimuli. To analyze the contextual memory, the mice were placed back into the conditioning chamber without a foot shock, and freezing behavior was recorded for 5 min. To analyze the cued memory, the mice were moved to a novel chamber without the steel rod for 3 min, followed by the administration of conditioning stimuli for 3 min. Separate groups of mice were used for contextual and cued memory analyses. The freezing behavior of the mice was recorded and analyzed. Short- and long-term memories were analyzed at 1 and 24 hr, respectively, after the initial fear conditioning test session.

Morris water maze

The Morris water maze test was performed as previously described[7,8], with modifications. Handling was performed daily (for 5 min) for five days prior to the experiment. Each mouse was subjected to a four-trial session for pre-training. Each mouse was placed on the visible platform for at least 15 sec, followed by a 60 sec swimming period. The mouse was released into the water at the water level and the releasing point of each trial was different. For spatial acquisition (training session), each mouse was trained four times per day with the hidden platform for five consecutive days. Before the first trial of the first session, each mouse was placed on the hidden platform for 15 sec. The mouse was subsequently released into the water for 60 sec and the escape latency was recorded. The recording was terminated when the mouse found the platform, where it would remain for 15 sec. To examine the reference memory, probe tests were performed on the third and fifth days of the training. The hidden platform was removed, and the mouse was allowed to swim in the pool for 60 sec. For the spatial reversal test, the hidden platform was relocated to the opposite quadrant. A new set of four trials was conducted each day for three additional days. The reversal probe trial was performed at the end of the reversal test. The swimming path, speed, and time spent in different quadrants by the mice were analyzed using a video tracking system, TrackMot (Singa Technology, Taiwan).

Electrophysiology

The brains of $AC6^{+/+}$ and $AC6^{-/-}$ mice (3-6 months old) were removed and chilled in ice-cold modified oxygenated artificial cerebrospinal fluid (ACSF) (87 mM NaCl, 25 mM $NaHCO_3$, 1.25 mM NaH_2PO_4, 2.5 mM KCl, 10 mM glucose, 75 mM sucrose, 0.5 mM $CaCl_2$, and 7 mM $MgCl_2$) after rapid decapitation. The mouse brains were was sectioned into transverse hippocampal slices with 250-350 μm thickness using a vibrating tissue slicer (DTK-1000; Dosaka, Kyoto, Japan) in ice-cold modified ACSF followed by incubation in the oxygenated (95% O_2, 5% CO_2) solution at 34°C for 25 min and stored at room temperature until further use as previously described[9,10]. During synaptic transmission experiments, the slices were transferred into a perfusion system equipped recording chamber that was filled with oxygenated ACSF (125 mM NaCl, 25 mM $NaHCO_3$, 1.25 mM NaH_2PO_4, 2.5 mM KCl, 25 mM glucose, 2 mM $CaCl_2$, and 1 mM $MgCl_2$). A monopolar stimulation electrode was placed on the Schaffer collateral pathway. A recording electrode was placed on the stratum radiatum for extracellular recording. To examine the NMDAR/AMPAR ratio, whole-cell voltage-clamp recordings were obtained from CA1 PCs. The intracellular pipette solution contained (in mM) 121.5 $CsMeSO_3$, 0.1 EGTA, 4 MgCl2, 13.5 CsCl, 10 HEPES, 5 QX-314 bromide, 2 Na_2ATP, 10 Na_2-phosphocreatine, 0.3 Na_3GTP, pH 7.3, adjusted with 1 M CsOH (290-300 mOsm). EPSCs were evoked every 15 s using a monopolar stimulation electrode filled with ACSF and placed in the stratum radiatum. The CA1 PCs were first voltage-clamped at -70 mV, and EPSCs were evoked at ~30% of maximum peak amplitude. After a stable 10-min baseline was established, the cells were voltage-clamped at +50 mV and EPSCs were recorded for 5 min. The NMDAR/AMPAR ratios were calculated by dividing the peak amplitude of an averaged AMPAR EPSC at -70 mV by the amplitude of an averaged EPSC at +50 mV at 50 ms after stimulation (NMDAR EPSC).

For field excitatory postsynaptic potential (fEPSP) recording, the $GABA_A$ receptor antagonist gabazine (1 μM) was applied in bath solution, and the stimulus was administered at 0.03 Hz. Stable baseline was recorded at least for 15 min before experiment. Long-term potentiation (LTP) were induced by applying two trains of high-frequency stimulation (HFS, each train includes 100 Hz for 1 s, train-to-train interval is 20 s) or theta burst stimulation (TBS, 5 bursts at 5 Hz, each burst includes 4 pulses at 100 Hz), and recorded for 1 hr.

For LTD experiments, after measurement of the input-output curve, the stimulus

intensity was adjusted to evoke 40 - 50% of the maximal fEPSP. After establishing a 15-min stable baseline (< 5% change), LTD was induced by applying low-frequency stimulation (LFS) or paired-pulse stimulation (PPS). LFS comprised 1800 pulses at 2 Hz (NMDAR-dependent LTD), whereas PPS comprised 900 pairs at 1 Hz with 50 ms (NMDAR-independent LTD) or 200 ms (NMDAR-dependent LTD) inter-pulse interval. The LTD level was determined from the averaged fEPSP slope recorded during 50-60 min after induction and normalized to the baseline. Ro25-6981 was bath applied at least 1 hr before LTD induction and throughout the entire experiment.

Supplemental Experimental Procedures References

1 Wang, S. C. *et al.* Regulation of type V adenylate cyclase by Ric8a, a guanine nucleotide exchange factor. *The Biochemical journal* **406**, 383-388, doi:10.1042/bj20070512 (2007).

2 Lai, H. L. *et al.* Protein kinase C inhibits adenylyl cyclase type VI activity during desensitization of the A2a-adenosine receptor-mediated cAMP response. *The Journal of biological chemistry* **272**, 4970-4977 (1997).

3 Shin, E. *et al.* Doublecortin-like kinase enhances dendritic remodelling and negatively regulates synapse maturation. *Nature communications* **4**, 1440, doi:10.1038/ncomms2443 (2013).

4 Wu, W. L., Lin, Y. W., Min, M. Y. & Chen, C. C. Mice lacking Asic3 show reduced anxiety-like behavior on the elevated plus maze and reduced aggression. *Genes, brain, and behavior* **9**, 603-614, doi:10.1111/j.1601-183X.2010.00591.x (2010).

5 Walf, A. A. & Frye, C. A. The use of the elevated plus maze as an assay of anxiety-related behavior in rodents. *Nat. Protocols* **2**, 322-328 (2007).

6 Sankoorikal, G. M. V., Kaercher, K. A., Boon, C. J., Lee, J. K. & Brodkin, E. S. A Mouse Model System for Genetic Analysis of Sociability: C57BL/6J Versus BALB/cJ Inbred Mouse Strains. *Biological Psychiatry* **59**, 415-423, doi:http://dx.doi.org/10.1016/j.biopsych.2005.07.026 (2006).

7 Malleret, G., Hen, R., Guillou, J. L., Segu, L. & Buhot, M. C. 5-HT1B receptor knock-out mice exhibit increased exploratory activity and enhanced spatial memory performance in the Morris water maze. *The Journal of neuroscience : the official journal of the Society for Neuroscience* **19**, 6157-6168 (1999).

8 Vorhees, C. V. & Williams, M. T. Morris water maze: procedures for

assessing spatial and related forms of learning and memory. *Nature protocols* **1**, 848-858, doi:10.1038/nprot.2006.116 (2006).

9 Lien, C. C., Martina, M., Schultz, J. H., Ehmke, H. & Jonas, P. Gating, modulation and subunit composition of voltage-gated K(+) channels in dendritic inhibitory interneurones of rat hippocampus. *The Journal of physiology* **538**, 405-419 (2002).

10 Lien, C. C. & Jonas, P. Kv3 potassium conductance is necessary and kinetically optimized for high-frequency action potential generation in hippocampal interneurons. *The Journal of neuroscience : the official journal of the Society for Neuroscience* **23**, 2058-2068 (2003)
